# *Cotton Leaf Curl Multan virus* C4 protein suppresses both transcriptional and post-transcriptional gene silencing by interacting with SAM synthetase

**DOI:** 10.1371/journal.ppat.1007282

**Published:** 2018-08-29

**Authors:** Asigul Ismayil, Yakupjan Haxim, Yunjing Wang, Huangai Li, Lichao Qian, Ting Han, Tianyuan Chen, Qi Jia, Alexander Yihao Liu, Songbiao Zhu, Haiteng Deng, Rena Gorovits, Yiguo Hong, Linda Hanley-Bowdoin, Yule Liu

**Affiliations:** 1 MOE Key Laboratory of Bioinformatics, Center for Plant Biology, Tsinghua-Peking Joint Center for Life Sciences, School of Life Sciences, Tsinghua University, Beijing, China; 2 MOE Key Laboratory of Bioinformatics and the Center of Biomedical Analysis, School of Life Sciences, Tsinghua University, Beijing, China; 3 Institute of Plant Sciences and Genetics in Agriculture, Robert H. Smith Faculty of Agriculture, Food and Environment, Hebrew University of Jerusalem, Rehovot, Israel; 4 Research Centre for Plant RNA Signaling, College of Life and Environmental Sciences, Hangzhou Normal University, Hangzhou, China; 5 Department of Plant and Microbial Biology, North Carolina State University, Raleigh, North Carolina, United States of America; University of California, Davis Genome Center, UNITED STATES

## Abstract

Gene silencing is a natural antiviral defense mechanism in plants. For effective infection, plant viruses encode viral silencing suppressors to counter this plant antiviral response. The geminivirus-encoded C4 protein has been identified as a gene silencing suppressor, but the underlying mechanism of action has not been characterized. Here, we report that *Cotton Leaf Curl Multan virus* (CLCuMuV) C4 protein interacts with S-adenosyl methionine synthetase (SAMS), a core enzyme in the methyl cycle, and inhibits SAMS enzymatic activity. By contrast, an R13A mutation in C4 abolished its capacity to interact with SAMS and to suppress SAMS enzymatic activity. Overexpression of wild-type C4, but not mutant C4^R13A^, suppresses both transcriptional gene silencing (TGS) and post-transcriptional gene silencing (PTGS). Plants infected with CLCuMuV carrying C4^R13A^ show decreased levels of symptoms and viral DNA accumulation associated with enhanced viral DNA methylation. Furthermore, silencing of *NbSAMS2* reduces both TGS and PTGS, but enhanced plant susceptibility to two geminiviruses CLCuMuV and *Tomato yellow leaf curl China virus*. These data suggest that CLCuMuV C4 suppresses both TGS and PTGS by inhibiting SAMS activity to enhance CLCuMuV infection in plants.

## Author summary

Geminiviruses are single-stranded DNA (ssDNA) viruses that infect a wide range of plant species and are responsible for substantial crop damage worldwide. However, how geminiviruses inhibit plant antiviral gene silencing defense is unclear. Here, we report that a single geminiviral protein CLCuMuV C4 inhibits both plant transcriptional gene silencing (TGS) and post-transcriptional gene silencing (PTGS) to promote an effective viral infection. We show that CLCuMuV C4 protein interacts with SAMS, a core enzyme in methyl cycle, and inhibits SAMS activity. Overexpression of CLCuMuV C4 reduces the DNA methylation levels of both a transgene and an endogenous locus. Further, silencing of SAMS reduced both TGS and PTGS, and enhanced viral infection while CLCuMuV virus carrying a mutation in C4 that fails to interact with SAMS showed decreased infection. These findings reveal a novel mechanism by which the CLCuMuV C4 protein suppress SAMS mediated TGS and PTGS, leading to enhanced viral infection in plant.

## Introduction

In the course of plant-virus interactions, plants have evolved ingenious counter-attack mechanisms to diminish or eliminate invading viral pathogens. Of various plant antiviral defenses, gene silencing can target either viral RNAs for degradation through post-transcriptional gene silencing (PTGS) or DNA sequences of DNA viruses for epigenetic modification through transcriptional gene silencing (TGS) [1,2].

DNA cytosine methylation is an important epigenetic marker for gene silencing and controls plant development and gene expression. It also plays an important role in plant defense against invading DNA viruses [3–5]. Cytosine nucleotides are methylated at the 5′ position of the pyrimidine ring to generate 5-methyl cytosine (5-mC), which is catalyzed by cytosine methyl transferases. In addition, PTGS also requires small RNA methylation [6]. In PTGS, the methyltransferase HEN1 adds a 2’-O-methyl group to the 3’-terminal nucleotide of small RNAs to protect them from a 3’-end uridylation activity [7]. S-adenosylmethionine (SAM) is the universal methyl group donor in DNA or RNA methylation, and its metabolic regeneration through methyl cycle involves four reactions: (1) SAM demethylation to generate S-Adenosyl-L-homocysteine (SAH) by SAM methyltransferases, (2) SAH hydrolysis to produce homocysteine by SAH hydrolase (SAHH), (3) methylation of homocysteine to yield methionine by methionine synthase, and (4) adenylation of methionine to form SAM by SAMS (S-adenosylmethionine synthase, MetK, MAT) [8]. Several publications have proposed that some DNA viruses can interfere with the proper function of the methyl cycle to reduce plant DNA methylation for effective infection [9–12].

Geminiviruses are a large family of plant viruses with small circular, single-stranded DNA genomes. They infect a broad range of plants and are classified into seven genera (Becurtovirus, Begomovirus, Curtovirus, Eragrovirus, Mastrevirus, Topocuvirus and Turncurtovirus) according to the viral genome organization, insect vector and host range [13]. Begomoviruses are transmitted by whiteflies and possess monopartite or bipartite genomes, the latter designated as DNA-A and DNA-B. Many monopartite begomoviruses are associated with satellites [14]. For example, *Cotton Leaf Curl Multan virus* (CLCuMuV) is an important monopartite begomovirus and infects many plant species including cotton and *N*. *benthamiana*. CLCuMuV can cause leaf curl disease, a devastating disease of cotton when it is associated with a betasatellite DNA.

To effectively infect plants, geminiviruses often need to interfere with PTGS or TGS pathways. Viral proteins such as AC2/C2, AC4/C4, V2, AC5/C5 encoded by different geminiviruses and *β*C1 protein encoded by viral satellite DNA are capable of inhibiting various steps in the PTGS pathway [15–20]. Other geminiviral proteins including C2/AC2 of *Beet curly top virus*, *Beet severe curly top virus* (BSCTV), *Tomato golden mosaic virus* and *Cabbage leaf curl virus* [9,10,12,21], βC1 of *Tomato yellow leaf curl China virus* betasatellite (TYLCCNB), C4 of *Tomato leaf curl Yunnan virus* [11,22], Rep of *Tomato yellow leaf curl Sardinia virus* (TYLCSV) and V2 of *Tomato yellow leaf curl virus* (TYLCV) [23,24] are identified as suppressors of TGS.

Geminivirus *AC4/C4* gene overlaps entirely within the replication initiation protein (Rep) coding region, but is in a different open reading frame. In the bipartite begomoviruses, *African cassava mosaic virus* and *Sri Lankan cassava mosaic virus*, AC4 suppresses PTGS by binding to miRNAs and siRNAs [15,25,26]. C4 affects the movement of some curtoviruses and monopartite begomoviruses [27–29] and acts as a cell cycle regulator [30]. Geminiviral C4 is one of viral symptom determinants [31–33], and functions as viral suppressor of RNA silencing for several bipartite and monopartite geminiviruses. Transgenic expression of curtovirus *C4* gene induces hyperplasia and alters plant development in *N*. *benthamiana* and Arabidopsis. It has been reported that geminiviral C4 interacts with host factors including multiple plant shaggy-like kinases [33–38]. The C4 protein from *Tomato yellow leaf curl virus* can inhibit the intercellular spread of RNAi [39]. These observations reveal the multifunctional nature of the C4 protein. CLCuMuV C4 also suppresses PTGS [40], but the underlying mechanism of PTGS suppression by C4 is still unknown.

In this study, we report that CLCuMuV C4 protein is able to suppress both TGS and PTGS by interacting with and inhibiting NbSAMS2, a novel geminiviral target. Further, silencing of *NbSAMS2* suppresses both TGS and PTGS and enhances CLCuMuV and TYLCCNV infection, providing direct evidence that interfering with a key enzyme in methyl cycle can promote geminiviral infection. These results provide compelling evidence of the requirement of a functional methyl cycle for both TGS-based and PTGS-based anti-geminiviral defense in plants. Disruption of this process by C4 contributes to successful establishment of geminivirus infection.

## Results

### NbSAMS2 is a C4-interacting protein

To identify CLCuMuV C4 interacting proteins, we employed GFP Trap coupled with mass spectrometry analysis. GFP-tagged CLCuMuV C4 (C4-GFP) was expressed transiently in *N*. *benthamiana* leaves. The total protein extracts were incubated with GFP-Trap_A beads (ChromoTek). After washing, the purified GFP-tagged C4 proteins were denatured at 98°C, separated on a SDS-PAGE gel (12%), and visualized by silver staining (S1A Fig). When the Liquid Chromatography with Mass Spectrometric (LC-MS/MS) polypeptide profiles were searched against *N*. *benthamiana* protein databases, a S-adenosyl methionine synthesase 2 (NbSAMS2) partial sequence was found with high scores (FVIGGPHGDAGLTGR) (S1B Fig). At the next stage, we searched the *N*. *benthamiana* genome database (http://solgenomics.net) using NbSAMS2, and found eight SAMS homologs. However, we were only able to clone the cDNAs of *NbSAMS1*, *NbSAMS2* and *NbSAMS3*, suggesting that the other five genes are not expressed or poorly expressed in leaf tissues. This finding is consistent with the RNA-seq database (http://benthgenome.qut.edu.au/), which shows the reads for *NbSAMS1*, *NbSAMS2* and *NbSAMS3* are 147, 858 and 87, respectively, but 0 or below 40 reads for the other *NbSAMS* homologues (We refer to these SAMS homologues collectively as *NbSAMS*). NbSAMS2 shares 90.7% and 91.5% amino acid identity with NbSAMS1 and NbSAMS3, respectively (S2 Fig).

### C4 protein interacts with NbSAMS *in vitro* and *in vivo*

To verify the interaction of C4 with NbSAMS, we first employed Firefly Luciferase Complementation Imaging assay [41]. Three different *NbSAMSs* were fused to the C-terminal domain of luciferase (cLUC) to generate cLUC -NbSAMS1, cLUC-NbSAMS2 and cLUC-NbSAMS3, and C4 was fused to the N-terminal domain of luciferase (nLUC) to generate C4-nLUC. C4-nLUC was co-expressed with cLUC-NbSAMS1, cLUC-NbSAMS2 or cLUC-NbSAMS3 in *N*. *benthamiana*. Chemical signals were detected when C4 was combined with all three NbSAMSs, due to the reconstitution of the luciferase activity by C4-NbSAMS interactions. However, no interaction was detected in negative controls (S3A Fig). These data suggest that C4 interacts with all three NbSAMSs. Considering very high amino acid identities among the three NbSAMS homologues and its highest expression level in leaf tissues, we focused on NbSAMS2 for subsequent analyses.

**Fig 1:**
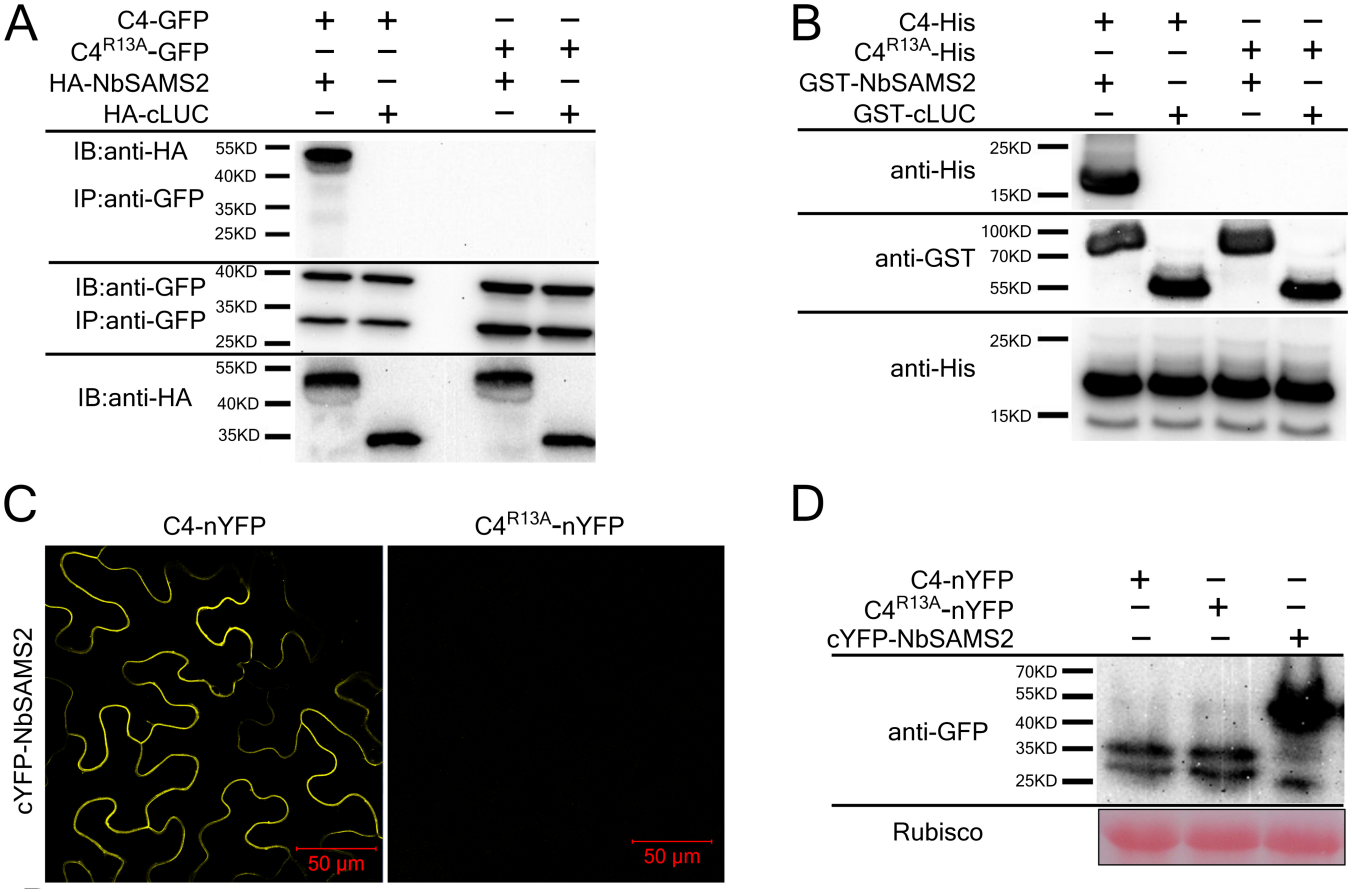
CLCuMuV C4 protein interacts with NbSAMS2 *in vitro* and *in vivo*. **(A)** CLCuMuV C4 protein co-immunoprecipitated with NbSAMS2. Total protein extracts were immunoprecipitated with anti-GFP beads and then monitored by immunoblotting (IB) using anti-GFP or anti-HA antibodies. cLUC represents c-terminal fragment of the firefly luciferase. **(B)** GST pull-down assay showed the interaction between C4 and NbSAMS2. Total soluble proteins of *E*. *coli* expressing GST-NbSAMS2 or GST were incubated with C4- His or C4^R13A^-His immobilized on glutathione-sepharose beads and monitored by anti-His antibody. **(C)** BiFC assay showed the interaction between C4 and NbSAMS2. Cells were photographed 60 hpi using confocal laser scanning microscope. Bar scale represents 50 μm. **(D)** Western blot analyses of BiFC construct combinations from the same experiments as in (C). All combinations were detected with anti-GFP polyclonal antibody.

The interaction between C4 and NbSAMS2 was further confirmed by co-immunoprecipitation (co-IP) assays. We found that C4-GFP protein co-immunoprecipitated with HA-NbSAMS2 but not with the control protein, HA-cLUC (Fig 1A). Further, C4 amino acids 1 to 20 are critical for interaction with NbSAMS2 (S3B Fig). Moreover, co-IP assays, GST pull-down assays and BiFC assays revealed that mutation of residue 13 from arginine to alanine (C4^R13A^) eliminates the interaction between C4 and NbSAMS2 (Fig 1). Several other C4 mutants (A3L, I5A, S6A, C8A, R13K, A14G) still interact with SAMS. It is worthwhile mentioning that the R13A mutation in C4 does not change the subcellular localization of C4 (S3D Fig).

To examine whether C4 interacts directly with NbSAMS2 *in vitro*, we performed glutathione S-transferase (GST) pull-down assays. 6×His-tagged C4 (C4-His) or C4^R13A^ (C4^R13A^-His) was expressed in *E*. *coli* BL21 (DE3) and purified on a Ni-NTA agarose column. After elution, C4-His and C4^R13A^-His were incubated with GST-tagged NbSAMS2 (GST-NbSAMS2) or GST-cLUC, respectively. C4-His, but not C4^R13A^-His, was pulled down with GST-NbSAMS2, but not GST-cLUC, suggesting that C4 directly interact with NbSAMS2 (Fig 1B).

C4-NbSAMS2 interaction was also examined using citrine yellow fluorescent protein (YFP)-based bimolecular fluorescence complementation (BiFC) assays [42]. Only when C4-nYFP was coexpressed with cYFP-NbSAMS2, strong YFP fluorescence was visible in cytoplasm in *N*. *benthamiana* leaf cells, whereas no YFP fluorescence was detected when C4^R13A^-nYFP was coexpressed with cYFP-NbSAMS2 (Fig 1C) or negative controls (S4A Fig). Immunoblot assays showed that all constructs were successfully expressed (Fig 1D).

Taken together, our results demonstrate that C4 interacts with NbSAMSs, and the R13 residue is essential for the C4-NbSAMS2 interaction.

### CLCuMuV C4 protein inhibits NbSAMS2 enzyme activity

**Fig 2:**
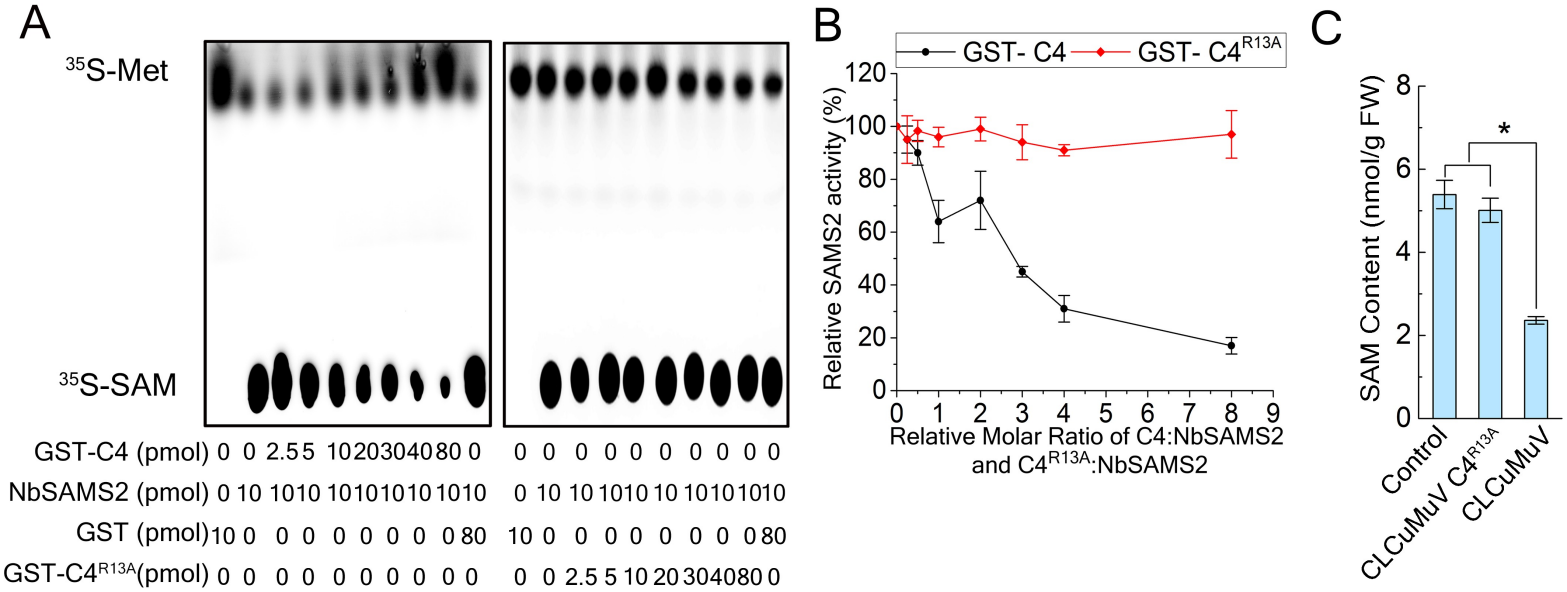
C4 protein inhibits NbSAMS2 activity. **(A)** Autoradiograph of a chromatogram analysis. The production of SAM was catalyzed by SAMS in reactions that containing varying amount of GST-C4 (or GST-C4^R13A^). **(B)** Quantification of relative SAMS activity. Data were obtained from three independent experiments. Bar represent means ± SE. **(C)** SAM content in Control (non-infection), CLCuMuV or CLCuMuV C4^R13A^ infected *N*. *benthamiana* plants. Values represent means ± SE from three independent experiments. (*p<0.05).

Given that C4 interacts with SAMS, it may affect SAMS enzymatic activity. To address this hypothesis, we performed an *in vitro* assay to test the effect of C4 on NbSAMS2 enzymatic activity by detecting amount of ^35^S-SAM in the presence of C4 or its mutant. For this, we expressed the fusion proteins GST-C4, GST-C4^R13A^ and GST-NbSAMS2 in *E*.*coli* and purified the proteins using glutathione sepharose. NbSAMS2 was pre-incubated for 20 minutes with varying amounts of GST-C4, GST-C4^R13A^ or GST. The protein mixtures were then added to solutions containing ^35^S-Met, dATP and MgCl_2_. After incubation at 30°C for 20 minutes, conversion of methionine to SAM was blocked by the addition of EDTA and monitored by thin layer chromatography (TLC). As expected, GST-NbSAMS2 efficiently converted methionine to SAM, showing a strong signal on TLC, but GST alone did not give any signal. Intriguingly, GST-C4 inhibited NbSAMS2 activity and maximal inhibition with approximately an 80% reduction were detected at an 8:1 molar ratio of C4:NbSAMS2. NbSAMS2 activity was not influenced by GST-C4^R13A^ or free GST (Fig 2). These results showed that C4 inhibits the enzymatic activity of NbSAMS2 through physical interaction.

C4 but not C4^R13A^ inhibits the enzymatic activity of NbSAMS2. Thus, we asked whether infection of CLCuMuV or CLCuMuV mutant virus (CLCuMuV-C4^R13A^, replacing C4 with its mutant counterpart C4^R13A^) affect the enzymatic activity of NbSAMS2 and influence the synthesis of SAM. *N*. *benthamiana* leaves were inoculated with CLCuMuV or CLCuMuV-C4^R13A^. At 21 days post inoculation (dpi), SAM was extracted from systemically infected leaves with 5% (w/v) trichloroacetic acid, and its level was analysed by LC-MS/MS [43]. CLCuMuV infected plants showed a reduced level of SAM (2.4 nmol/g) compared to CLCuMuV-C4^R13A^ (4.9 nmol/g) and healthy control plants (5.6 nmol/g) (Fig 2C). Taken together, these results showed that CLCuMuV C4 protein inhibits NbSAMS2 enzyme activity by interacting with NbSAMS2.

### SAMS is involved in both TGS and PTGS in plants

**Fig 3:**
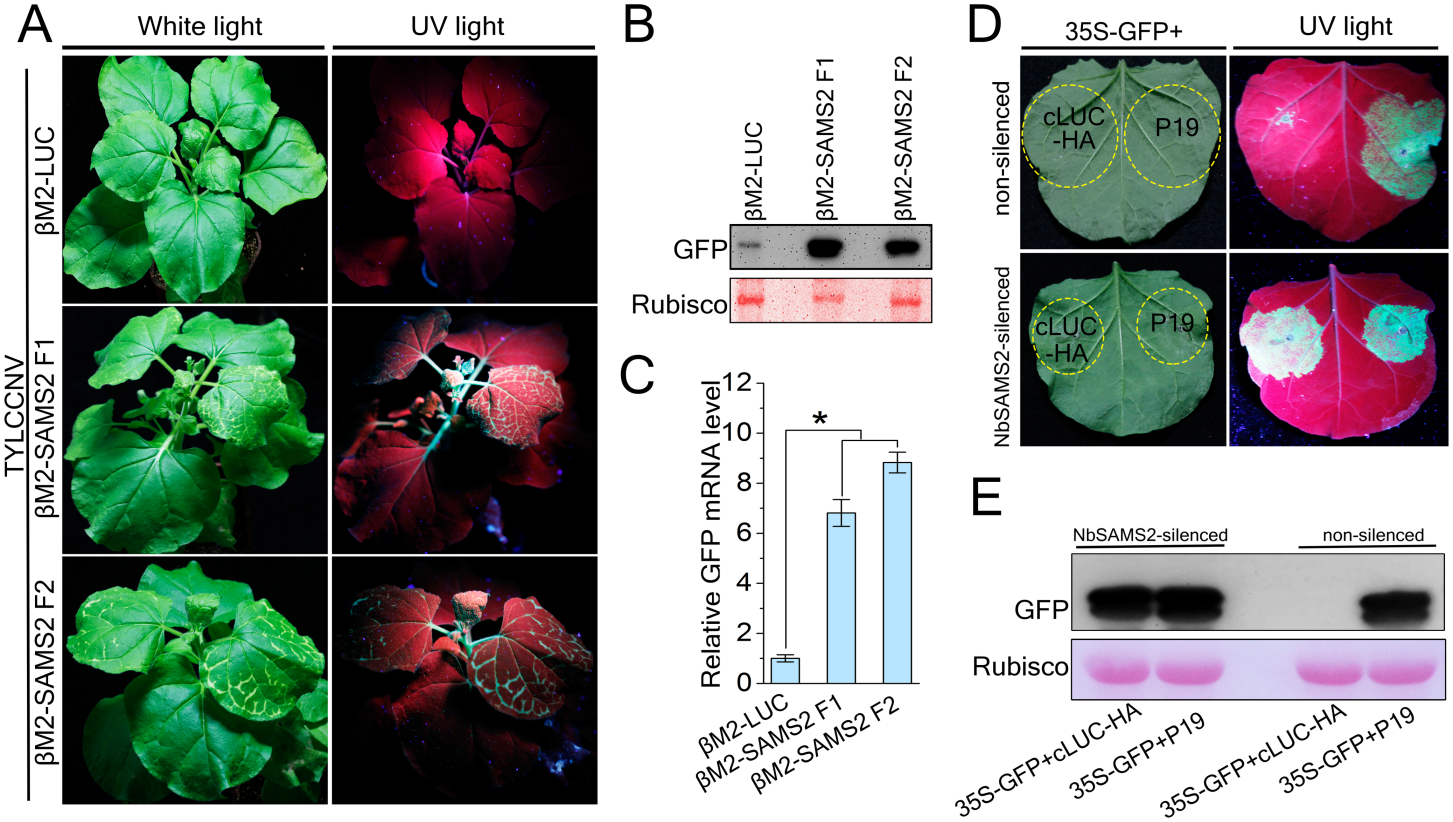
Silencing of *NbSAMS2* reverses TGS and PTGS of GFP. **(A)**16-TGS plants were co-inoculated with TYLCCNV and βM2 vector containing DNA fragment of *NbSAMS2* or LUC. Plants were photographed under UV light at 21 dpi. **(B)** Western blot assay of GFP accumulation in inoculated plants. GFP protein level was assessed by anti-GFP antibody. Ponceau Red Stained Rubisco was used as an equal protein loading control. **(C)** Real-time RT-PCR showed relative GFP mRNA levels of leaves of 16-TGS plants inoculated as indicated. Data were obtained from three independent experiments. Values represent means ± SE from three independent experiments. (*p<0.05). **(D)** GFP fluorescence in leaves of *N*. *benthamiana* plants co-infiltrated 35S-GFP together with indicated suppressor. **(E)** Western blot assay of GFP accumulation in inoculated plants shown in (**D**). GFP protein level was assessed by anti-GFP antibody. Ponceau Red Stained Rubisco was used as a protein loading control.

Given that SAMS is a key enzyme that converts methionine to SAM, a methyl donor for methylation of RNA, proteins and DNA [44], it may be involved in gene silencing in plants. To test the role of SAMS in TGS pathway, we cloned two 345-bp *NbSAMS2* fragment (at 3′ or 5′ UTR) and a 345-bp luciferase fragment (LUC) into CLCuMuB-based VIGS vector βM2 [45] to generate βM2-SAMS2F1, βM2-SAMS2F2 and βM2-LUC (a negative control), respectively. We performed a heterologous VIGS assay using TYLCCNV and βM2 to silence *NbSAMS2* in 16c-TGS plants, because CLCuMuV encodes a TGS suppressor (e.g. C4 in this study) and TYLCCNV does not suppress TGS [11]. 16c-TGS plants contain a transcriptionally silenced GFP transgene flanked by the 35S promoter [2,21]. TYLCCNV supports the replication of βM2 (S4 Fig), indicating that the βM2 vector can be used to silence a plant gene when co-agroinfiltrated with TYLCCNV. Indeed, *NbSAMS2* mRNA levels were reduced ~ 50% in Nb*SAMS2*-silenced 16c-TGS plants compared to control plants (S5 Fig). As expected, no GFP fluorescence was seen in control plants. However, GFP fluorescence was visible in Nb*SAMS2* silenced 16c-TGS plants (Fig 3A). GFP expression was confirmed by immunoblot assays in Nb*SAMS2*-silenced but not in control 16c-TGS plants (Fig 3B). The relative level of GFP mRNA in Nb*SAMS2*-silenced plants was 7 to 9 times more than in the controls (Fig 3C). Further, silencing of *NbSAMS2* was associated with earlier symptom appearance and enhanced TYLCCNV DNA accumulation (4 to 6 times) (S5D Fig).

To test the role of SAMS in the PTGS pathway, we silenced *NbSAMS2* in *N*. *benthamiana* using *Tobacco rattle virus* (TRV)-based virus-induced gene silencing (VIGS) [46]. SAMS2-silenced plants showed albino and dwarf phenotypes 3–4 weeks post inoculation (S6 Fig), perhaps because TRV induced stronger VIGS of SAMS2 than CLCuMuB-based VIGS of SAMS2 (approximately 90% vs 55%, also see below). However, we were able to perform PTGS assays 2 weeks post inoculation. *NbSAMS2*-silenced plants were co-agroinfiltrated with a binary construct expressing GFP (35S-GFP) [47] and a binary construct expressing the HA-tagged C-terminal domain of luciferase (cLUC-HA) or P19-silencing suppressor from *Tomato bushy stunt virus* (P19) [48]. Five days after inoculation, co-expression of 35S-GFP and TBSV P19 gave GFP fluorescence in both non-silenced and Nb*SAMS2*-silenced plants (Fig 3D). However, co-expression of 35S-GFP and cLUC-HA gave GFP fluorescence in *NbSAMS2*-silenced plants, but not in non-silenced plants. GFP expression was confirmed by immunoblot analysis (Fig 3E). The level of *NbSAMS2* mRNA was reduced in Nb*SAMS2*-silenced *N*. *benthamiana* plants (S7 Fig).

These results suggest that *NbSAMS2* is required for both TGS and PTGS in plants, and involved in antiviral defense against TYLCCNV.

### Silencing of *SAMS* enhances CLCuMuV DNA accumulation

**Fig 4:**
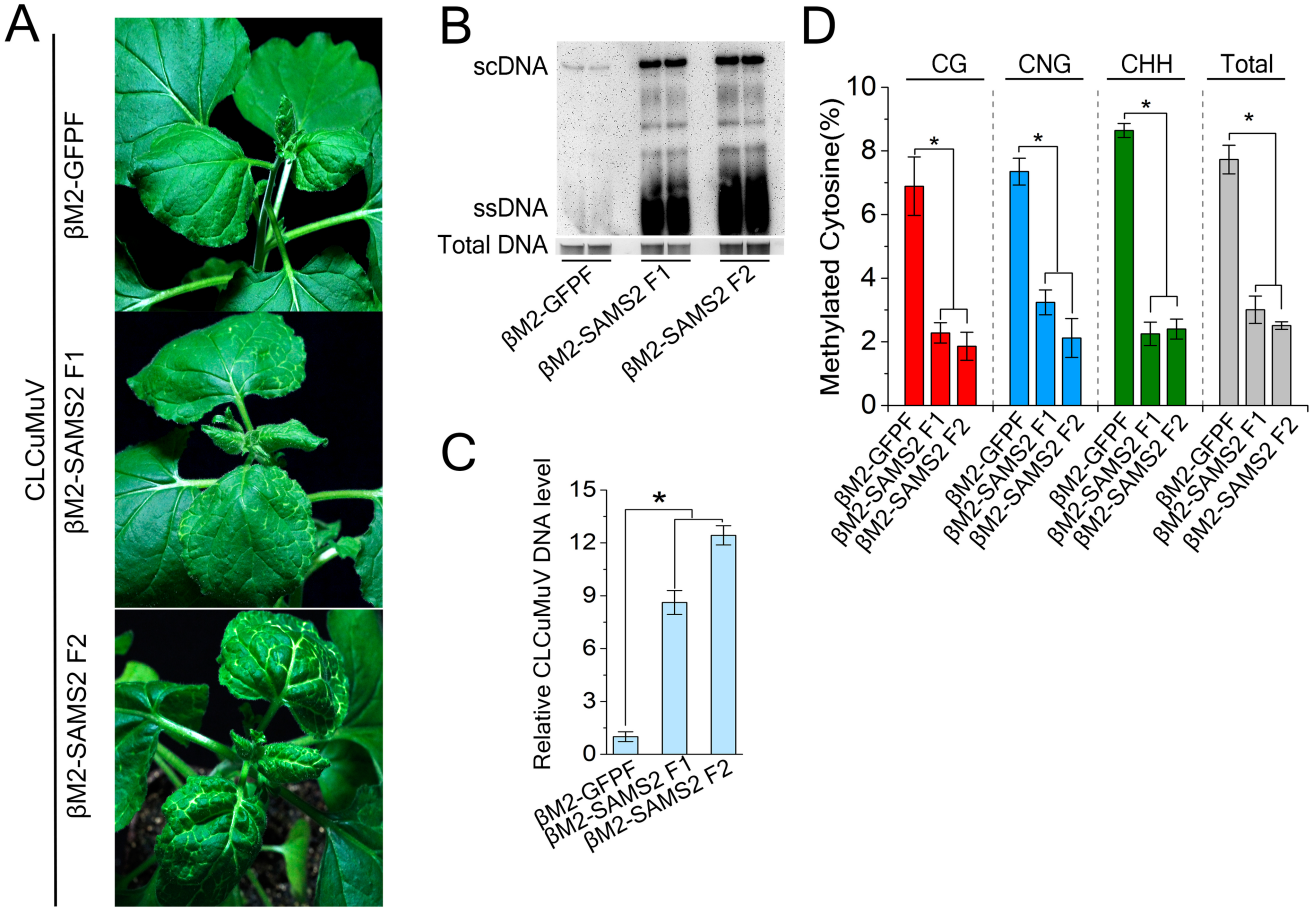
Silencing of *NbSAMS2* enhance plant susceptibility against CLCuMuV infection. **(A)** Symptom of *NbSAMS2*-silenced or control plants at 14 dpi. *N*. *benthamiana* plants were co-inoculated with CLCuMuV and its beta satellite VIGS vector containing DNA fragment of *NbSAMS2* or *GFP*. **(B)** Southern blot analysis of viral DNAs in CLCuMuV-infected plants shown in (**A**). Total DNAs were blotted with biotin-labeled probes specific for CLCuMuV V1. The DNA agarose gel was stained with ethidium bromide as a loading control. Viral single-stranded DNA (ssDNA) and supercoiled DNA (scDNA) are indicated. **(C)** Silencing of *NbSAMS2* increased viral DNA accumulation. Real-time PCR analysis of *V1* gene from CLCuMuV was used to determine viral DNA level. Values represent means ± SE from three independent experiments. (*p<0.05). **(D)** Silencing of NbSAMS2 reduced the cytosine methylation level of intergenic region of CLCuMuV. The histogram represents the percentage of total cytosine sites methylated in intergenic region of CLCuMuV in NbSAMS2-silenced or control plants. 15 individual clones were sequenced. N represents any nucleotide, and H represents A, T, or C. Values represent means ± SE. (*p<0.05).

To assess how C4-NbSAMS2 interaction contributes to CLCuMuV infection, we silenced *NbSAMS2* in *N*. *benthamiana* using CLCuMuB-based VIGS [45], because the transgenic knockdown of SAMS [49] and TRV-mediated silencing of SAMS2 (S6 Fig) cause a strong abnormal developmental phenotype. For this purpose, *N*. *benthamiana* plants were infected with CLCuMuV plus βM2-SAMS2F1, βM2-SAMS2F2 or negative control βM2-GFPF containing 345-bp GFP fragment [45]. Both control and *SAMS*-silenced plants showed a leaf curl symptom. However, viral symptom is much more severe in *SAMS2*-silenced plants. We observed some whitening veins in *SAMS*-silenced plants, suggesting a possible involvement of *SAMS* in pigment synthesis (Fig 4A). Total DNA was extracted from systemic leaves and analyzed DNA gel blots with biotin-labeled probes specific for CLCuMuV. Results indicated that CLCuMuV DNA accumulation significantly increased in *SAMS*-silenced plants compared to control plants (Fig 4B). CLCuMuV genomic DNA levels were measured by real time PCR using *eIF4a* as an internal control. The relative CLCuMuV DNA level in *SAMS2*-silenced plants was 9 to 12-fold compared to the non-silenced controls (Fig 4C). Real-time RT-PCR showed that silencing of *SAMS* using either βM2-SAMS2F1 or βM2-SAMS2F2 significantly reduced mRNA level of *NbSAMS2* (50% or 60%, respectively), but not of *NbSAMS3* (S8 Fig). In addition, silencing of *SAMS* using βM2-SAMS2F2, but not βM2-SAMS2F1, also reduced mRNA level of *NbSAMS1* (40%) (S8 Fig). Silencing of *SAMS2* show weak or no silencing of *SAMS1* and *SAMS3* because of the use of 3′ or 5′ UTR fragment of *NbSAMS2* to specifically silence *SAMS2*. RNA-seq data (https://solgenomics.net/) indicated that the expression levels of *SAMS1* and *SAMS3* are very low compared to *SAMS2*.

We examined the effect of silencing of *SAMS2* on the methylation status of CLCuMuV genomic DNA by bisulfite sequencing of the viral 5’-intergenic region (IR). This region contains 52 potential methylation sites, including nine CG sites, seven CNG sites, and 36 CHH sites. Cytosine methylation levels of the CLCuMuMV IR in *N*. *benthamiana* plants infected with CLCuMuV plus βM2-SAMS2F1, βM2-SAMS2F2 or the negative control βM2-GFPF are summarized in Fig 4A. The methylation level of the CLCuMuV IR is much lower in *SAMS*-silenced plants (20%) than the control plants (80%) (Fig 4D). Silencing of *SAMS2* caused strong suppression of DNA methylation.

Taken together, these results suggest that NbSAMS2 takes part in plant antiviral defenses against CLCuMuV by positively contributing to both TGS and PTGS.

### CLCuMuV C4 suppresses both TGS and PTGS pathways in plants

**Fig 5:**
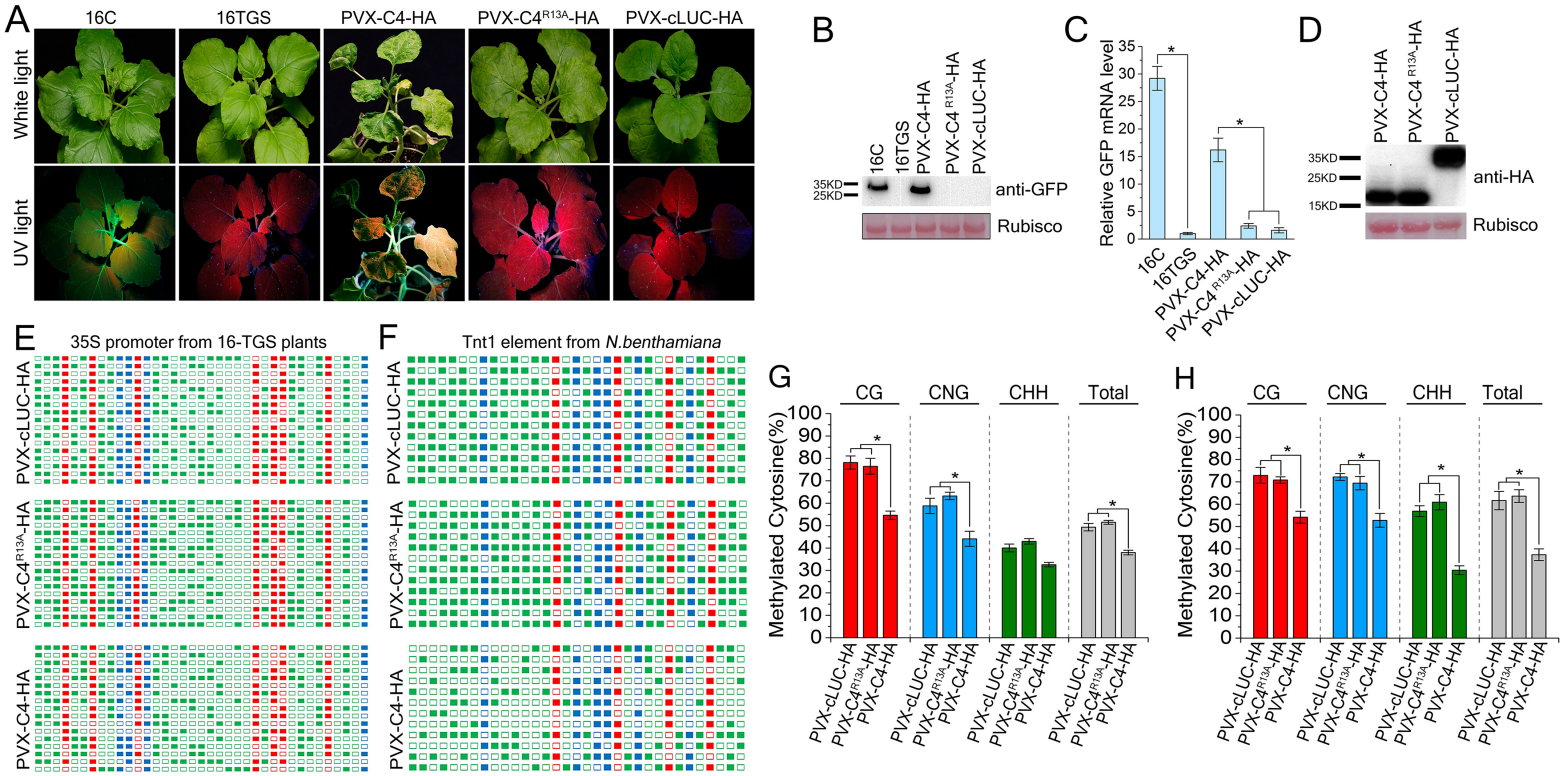
PVX-based expression of C4 reverses TGS of a GFP transgene and suppresses endogenous and exogenous DNA methylation. **(A)**16-TGS plants were inoculated with PVX-C4-HA, PVX-C4^R13A^-HA or PVX-cLUC-HA, and photographed under UV light at 14 dpi. cLUC represents c-terminal fragment of the firefly luciferase. **(B)** Western blot assay of GFP accumulation in inoculated plants. GFP protein level was assessed by anti-GFP antibody. Ponceau Red Stained Rubisco was used as a protein loading control. **(C)** Real-time RT-PCR showed relative GFP mRNA levels of leaves of 16-TGS plants inoculated as indicated. Values represent means ± SE from three independent experiments. (*p<0.05). **(D)** Western blot assays of C4 construct combinations from the same experiments as in (A). Samples were detected with anti-HA polyclonal antibody. **(E and F)** Cytosine methylation profiles of 35S-GFP transgene **(E)** and *NbTnt1* transposable element **(F)** confirm that C4 inhibits methylation. The cytosine methylation levels of 35S promoter and *NbTnt1* were assessed by bisulfite sequencing. The cytosine residues were represented by rectangles (red for CG, blue for CNG, and green for CHH). The methylated cytosines were indicated by filled rectangles while non-methylated cytosines were indicated by blank rectangles. Each line represents the sequence of an individual clone. **(G and H)** Percentage of methylated cytosine of 35S-GFP transgene **(G)** and *NbTnt1* transposable element **(H)**. The histogram represents the proportions of cytosine residues methylated in different sequence contexts. The statistical analysis was performed using the OriginPro 8 program. The bars denote the SE of the means, asterisks are representing significantly different from each group (one-way analysis of variance, *p<0.05).

Given that C4 interacts with NbSAMS2 and inhibits its enzymatic activity, we assumed that C4 may suppress NbSAMS2-mediated TGS. For this, we tested whether *Potato virus X* (PVX)-based expression of C4 (PVX-C4-HA) can reverse GFP expression in 16c-TGS plants. PVX-C4-HA infected 16c-TGS plants showed chlorosis and up-curling symptoms in systemic leaves while PVX-cLUC-HA and PVX-C4^R13A^-HA control plants only showed mild viral symptoms. At 14 dpi, GFP fluorescence became visible in leaves, especially in phloem tissue of the PVX-C4-HA infected 16c-TGS plants, but not in PVX-C4^R13A^-HA and PVX-cLUC-HA plants (Fig 5A). The relative GFP mRNA level in PVX-C4-HA infected 16c-TGS plants was 15-fold of that in the PVX-cLUC-HA infected 16c-TGS plants. We verified this result at the GFP protein level inn immunoblotting assays (Fig 5).

Bisulfite sequencing was used to assess the level of TGS suppression in the PVX-C4-HA infected *N*. *benthamiana* 16c-TGS plants [3,9,11,50]. We analyzed cytosine methylation at seven CG, four CNG, and 26 CHH sites in the 35S promoter of the GFP transgene (Fig 5E). PVX-based expression of C4 reduced cytosine methylation at CG (23%), CNG (15%) and CHH (8%) sites compared to PVX-based expression of C4^R13A^ or control protein cLUC (Fig 5G). We also measured the DNA methylation level of *Tnt1* retrotransposon. Here, *Tnt1* was served as an endogenous epigenetic marker in *N*. *benthamiana*. C4 reduced *Tnt1* cytosine methylation at CG (19%), CHH (26%) and CNG (20%) sites compared to C4^R13A^ or control protein cLUC (Fig 5F and 5H). These results indicated that CLCuMuV C4, but not its mutant C4^R13A^, is able to reduce cytosine methylation of both an endogenous gene and a transgene in plants.

TYLCCNV co-infection with its betasatellite Y10β or the Y10β-encoded protein βC1 alone can reverse TGS [11]. To further confirm TGS suppression activity of CLCuMuV C4, we replaced βC1 of Y10β with CLCuMuV *C4* or 345 bp of luciferase fragment (LUC) to generate Y10mβ-C4 or Y10mβ-LUC, that were co-inoculated with TYLCCNV onto *N*. *benthamiana* 16c-TGS plants. At 15 dpi, TYCLCCNV plus Y10β caused severe symptoms and TYCLCCNV plus Y10mβ-LUC only caused very mild symptoms. Y10mβ-C4 induced stronger viral symptoms than Y10mβ-LUC, but weaker symptoms than Y10β, when co-infected with TYLCCNV. Further, GFP fluorescence was visible in phloem tissue and young leaves of 16c-TGS line infected with TYLCCNV plus either Y10mβ-C4 or Y10β, but not when infected with TYLCCNV plus Y10mβ-LUC (S9A Fig). GFP expression was confirmed on immunoblots (S9B Fig). Interestingly, CLCuMuV C4 enhanced TYLCCNV DNA accumulation (S9C Fig). These data suggest that CLCuMuV C4 can reverse TGS and impair plant antiviral defense against TYLCCNV.

NbSAMS2 is involved in both TGS and PTGS in plants and C4 is a PTGS suppressor. To test whether the interaction of CLCuMuV C4 with NbSAMS2 is required for C4 PTGS suppressor activity, we coexpressed 35S-GFP with cLUC-HA, C4-HA, C4^R13A^-HA or P19 in *N*. *benthamiana* leaves using agroinfiltration to trigger PTGS. Strong GFP fluorescence were observed in tissues co-expressing 35S-GFP with C4-HA or P19, but not in tissues co-expressing 35S-GFP with C4^R13A^-HA or cLUC-HA at 5 dpi (S10A Fig). The relative GFP mRNA level in presence of C4 was 9 times more than the controls (S10B Fig). We verified this result GFP mRNA and protein by real-time RT-PCR and immunoblotting (S10C Fig). RNA gel blot analysis detected GFP-specific siRNAs in leaves infiltrated with GFP plus cLUC-HA or C4R13A-HA, but not in those infiltrated with GFP plus P19 or C4-HA and non-infiltrated plants (S10D Fig). These data suggest that CLCuMuV C4 functions as a PTGS suppressor dependent on its interaction with SAMS.

Taken together, our results suggest that CLCuMuV C4 is able to inhibit methylation mediated TGS and PTGS. C4-NbSAMS interaction is essential for C4 suppressor activity of gene silencing.

### A R13A point mutation in C4 attenuates viral infection

**Fig 6:**
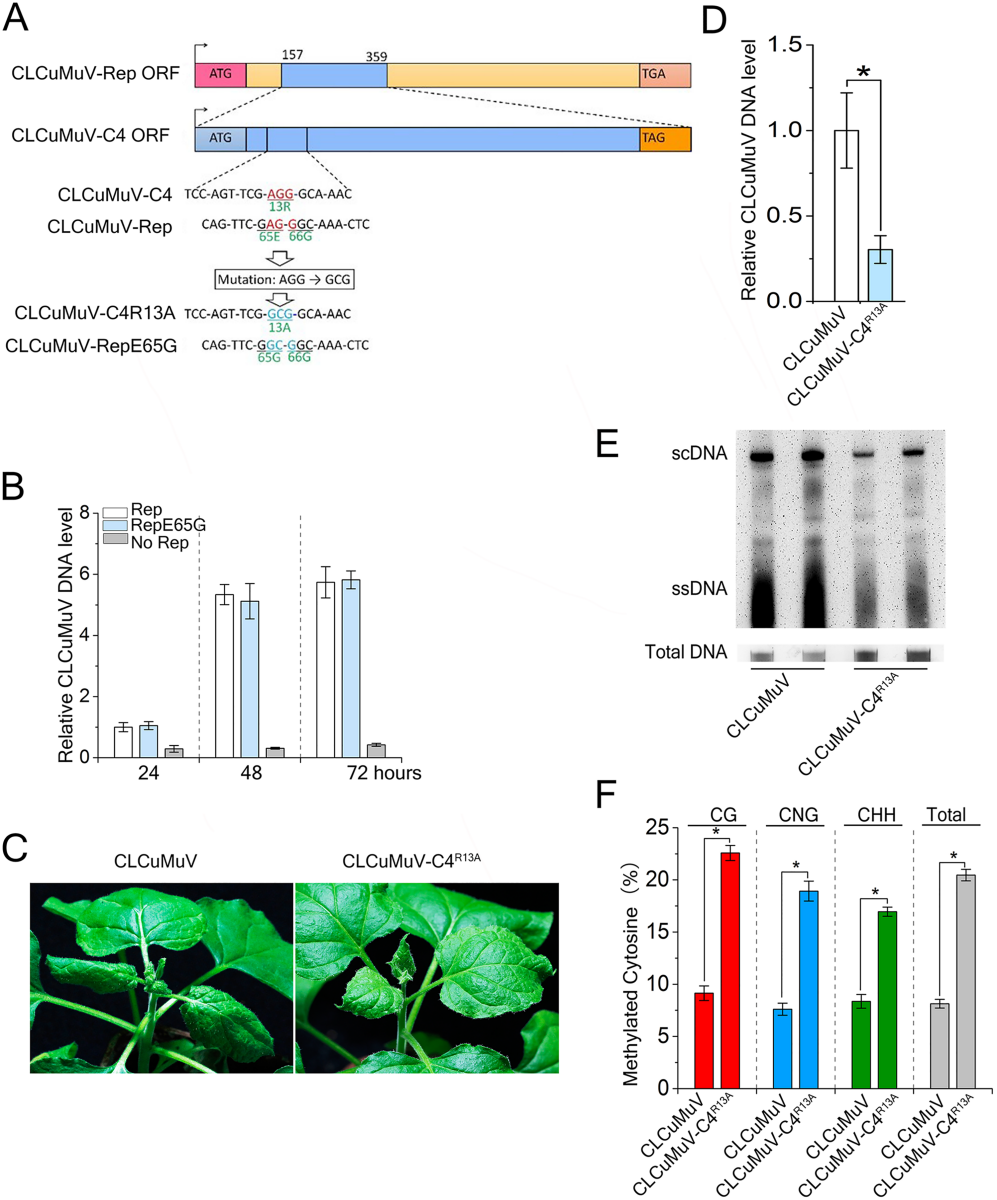
A R13A point mutation in C4 attenuates CLCuMuV infection. **(A)** Schematic representation of the CLCuMuV C4 and Rep ORF. Sequences in light blue color represents mutation while red color for original nucleotide and relevant amino acids was displayed with green color. **(B)** Relative viral accumulation of CLCuMuV DNA. Protoplasts from the leaves of *N*. *benthamiana* plants were isolated, and then transfected with CLCuMuVΔRep and expression construct of either Rep or RepE65G. Real-time PCR analysis of V1 gene from CLCuMuVΔRep was used to determine viral DNA level. *eIF4α* was used as an internal control. Values represent means ± SE from three independent experiments. **(C)** The mutant CLCuMuV carrying C4^R13A^ (CLCuMuV-C4^R13A^), which contains a R13A mutation in *C4*, showed the decreased viral symptom compared to wild type CLCuMuV. Photographs were taken at 21 dpi. **(D)** Relative viral accumulation of CLCuMuV DNA. Real-time PCR analysis of V1 gene from CLCuMuV was used to determine viral DNA level. Values represent means ± SE from three independent experiments. (*p<0.05). **(E)** Southern blot analysis of CLCuMuV accumulation in systemic leaves of plants shown in (**A**) at 21 dpi. Total DNAs were blotted with biotin-labeled probes specific for CLCuMuV V1. The DNA agarose gel was stained with ethidium bromide as a loading control. Viral single-stranded DNA (ssDNA) and supercoiled DNA (scDNA) are indicated. **(F)** Analysis of DNA Methylation of CLCuMuV and CLCuMuV-C4^R13A^ Genomes. The histogram shows the percentage of total cytosine sites methylated in the intergenic region of CLCuMuV and CLCuMuV-C4^R13A^ DNA isolated from infected *N*. *benthamiana* plants. 15 individual clones were sequenced. Values represent means ± SE. (*p<0.05).

Because the C4 gene entirely overlaps the Rep coding region, and the R13A mutation in the C4 protein resulted in a E65G mutation in the Rep protein (Fig 6A). To examine whether the E65G mutation in Rep (Rep^E65G^) impacts viral replication, we quantified DNA accumulation of a null mutant virus for the Rep gene, CLCuMuVΔRep (CLCuMuV with ATG-to-TGA mutation in start codon of Rep). For this, we isolated protoplasts from the leaves of *N*. *benthamiana* plants, and then transfected them with CLCuMuVΔRep and an expression construct for either Rep or Rep^E65G^. We extracted total DNA at 24, 48, 72 hours post transfection, and measured CLCuMuV DNA levels by real time PCR using *eIF4a* as an internal control. Real-time PCR analysis showed that viral DNA accumulation is similar between cells expressing Rep and Rep^E65G^ (Fig 6B). These results suggest that E65G mutation in Rep does not affect the function of Rep protein in viral replication, consistent with the fact that Rep protein of a natural CLCuMuV associated with Okra leaf curl disease has a G at its 65^th^ position (accession number ADD70021).

To further explore biological significance of the C4-NbSAMS2 interaction in CLCuMuV infection, we generated a CLCuMuV mutant virus (CLCuMuV-C4^R13A^) by replacing *C4* with its mutant counterpart *C4*^*R13A*^. *N*. *benthamiana* leaves were inoculated with CLCuMuV and CLCuMuV-C4^R13A^. At 15 dpi, viral DNA was detected in systemic leaves in both CLCuMuV and CLCuMuV-C4^R13A^ infected plants. At 21 dpi, CLCuMuV-C4^R13A^ caused an attenuated leaf curl symptom compared to wild-type CLCuMuV (Fig 6C), and viral DNA accumulation was reduced 3-fold in plants infected with CLCuMuV-C4^R13A^ compared to CLCuMuV (Fig 6D). This result was verified on DNA gel blots (Fig 6E). Because the R13A mutation in C4 abolished its interaction with SAMS and the E65G mutation in Rep had no effect on CLCuMuV replication, these results suggest that C4-NbSAMS2 interaction is important for viral infection. We hypothesize that C4 affects methylation of the viral DNA genome to enhance viral accumulation by interacting with and inhibiting SAMS2. To test this hypothesis, we examined the methylation status of the IR of CLCuMuV and CLCuMuV-C4^R13A^ by bisulfite sequencing. The results indicated that an R13A mutation in C4 enhanced IR methylation at CG (13%), CNG (11%) and CHH (8%) sites in plants (Fig 6F).

Taken together, our data suggest that CLCuMuV C4 contributes to viral infection by inhibiting SAMS-dependent methylation-mediated TGS and PTGS through its interaction with SAMS to inhibit SAMS enzyme activity in plants.

## Discussion

In this study, we showed that CLCuMuV C4 suppresses both TGS and PTGS by inhibiting SAMS activity through its interaction with SAMS in plants. Furthermore, a point mutation from arginine to alanine at position 13 in C4 abolishes the C4-NbSAMS2 interaction and C4-mediated TGS/PTGS suppression, resulting in impaired viral infection and enhanced viral DNA methylation. In addition, silencing of *NbSAMS2* reversed methylation-mediated TGS and PTGS and enhanced geminiviral infection. Our work provides direct evidence that a geminivirus is able to interfere with the methyl cycle to promote the effective infection, and reinforces the importance of methylation as an epigenetic defense against geminiviruses.

It is unclear how CLCuMuV C4 suppresses SAMS2 activity by interacting with SAMS. CLCuMuV C4 shares 78% identity and 85% similarity with *Cotton leaf curl Kokhran virus* (CLCuKoV) C4 at the protein level. CLCuMuV C4 could also have an ATPase activity like CLCuKoV C4 [51]. We found that CLCuMuV C4 R13 is required for its interaction with SAMS and its suppression of SAMS activity. Interestingly, CLCuKoV C4 R13 is involved in its ATPase activity [51]. Thus, CLCuMuV C4 may use its ATPase activity to suppress SAMS activity. Consistent with this idea, SAMS requires ATP for converting methionine to SAM [8].

We provided several lines of evidence to demonstrate the ability of the CLCuMuV C4 protein to inhibit DNA methylation-mediated TGS. First, both PVX-based and TYLCCNV-based expression of C4 reversed the transcriptionally silenced GFP transgene in *N*. *benthamiana* 16c-TGS plants. Second, expression of C4 reduced cytosine methylation of CaMV 35S promoter of the GFP transgene in 16c-TGS lines (Fig 5), suggesting that the demethylation in CaMV 35S promotor in 16c-TGS plants reactivates expression of the TGS-silenced GFP transgene. Third, expression of C4 reduced cytosine methylation of the *Tnt1* retrotransposon in *N*. *benthamiana*. Fourth, an R13A mutation in C4 enhanced geminiviral DNA methylation.

DNA methylation in plants can occur in different sequence contexts including symmetric (CG and CHG, where H is A, C, or T) sites and asymmetric (CHH) sites. In this study, we observed that silencing of *NbSAMS2* can reverse TGS of GFP transgene, and CLCuMuV C4 reduced cytosine methylation of CG and CNG in the transgene promoter region and CG, CHG, CHH in the retrotransposon *Tnt1* and the CLCuMuV genome. The C4-reduced methylation levels of Tnt1 and 35S promoter were different. The reason may be due to the difference in the methylation level of different loci. Indeed, The CHH methylation level of Tnt1 are higher than the methylation of 35S promoter, the reduction of methylation level of Tnt1 (CHH) was higher compared to the 35S promoter in the presence of C4. Further, CLCuMuV carrying C4^R13A^ showed increased viral DNA methylation in plants. Consistent with our observations, knockdown of *SAMS* effectively represses symmetric cytosine methylation in some key flowering genes [49].

In plants, the addition of a methyl group to DNA or RNA is thought to be one of the major host defense mechanisms against viruses [6,9]. Potyviral HCPro from Potato virus A (PVA) was reported to bind two methyl cycle-related proteins SAMS and SAHH. Further, knockdown of SAMS and SAHH partially rescues the HCPro-deficient PVA viral phenotype [52], suggesting that HC-Pro may suppress PTGS through disruption of the methyl cycle. However, the role of HC-Pro-SAMS interaction in PVA-mediated suppression of SAMS activity and PTGS has not been investigated. In addition, the symptoms of CLCuMuV in *N*. *benthamiana* are mild (Fig 6C). However, PVX-C4 exhibited severe symptoms in Fig 5A. The severe phenotype caused by PVX may be caused by the methyl cycle inhibition itself or synergistic effect of C4 on PVX, similar to effect of HC-Pro on PVX [52].

Plant DNA viruses have evolved different proteins to interfere with the plant methylation pathway. Adenosine kinase (ADK) is a cytoplasmic enzyme involved in the adenine and adenosine salvage pathways, and may have a role in sustaining the methyl cycle in both yeast and plants [53–55]. Geminiviral AC2/AL2 proteins negatively regulate TGS pathway by interacting with and inactivating ADK [9]. S-adenosyl-methionine decarboxylase 1 (SAMDC1) is a key enzyme for the synthesis of polyamines in mammals and plants [56]. BSCTV C2/L2 protein inhibits SAMDC1 activity [10]. TYLCCNV βC1 protein also represses cytosine methylation by interacting with S-adenosyl homocysteine hydrolase (SAHH), a methyl cycle enzyme required for SAM production and methylation-mediated TGS although it is not reported whether inhibiting SAHH affects geminivirus infection in plants [11]. These studies suggest that geminiviruses may disturb the proper function of the cellular methyl cycle to affect TGS. In our study, we demonstrate that CLCuMuV C4 impairs host methylation by directly binding to SAMS and inhibiting its enzyme activity in SAM production. The R13A mutation abolished ability of C4 to inhibit SAMS enzyme activity and to suppress TGS and PTGS. Further, CLCuMuV-C4^R13A^ has less infectious (Fig 6), suggesting that CLCuMuV C4 suppresses SAMS enzyme activity and gene silencing to enhance geminivirus infection. More importantly, silencing of *NbSAMS2* reversed TGS and PTGS and reduced plant resistance against two geminiviruses CLCuMuV and TYLCCNV (Figs 3 and 4 and S5 Fig), providing direct evidence that a geminivirus-encoded protein is able to promote virus infection by interfering with SAMS-mediated methylation-dependent TGS and PTGS.

**Fig 7:**
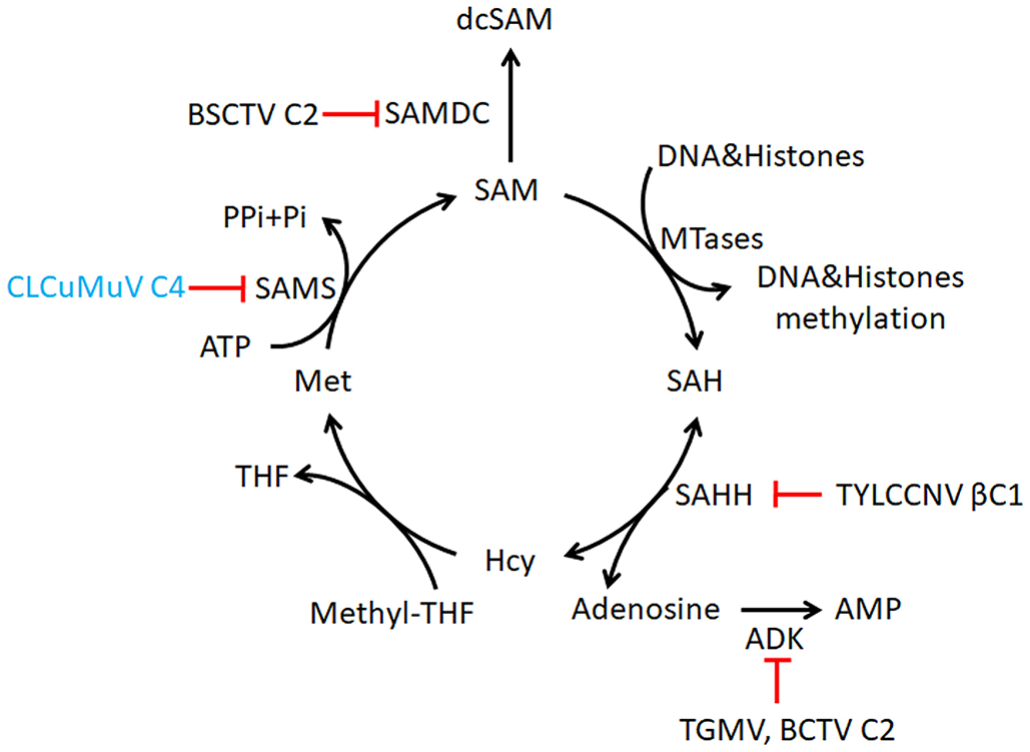
Model for methyl cycle inactivation by geminiviral proteins. In this figure, Met for L-methionine; SMA for S-adenosyl methionine; SAH for S-adenosyl homocysteine; Hcy for homocysteine; THF for tetrahydrofolate; PPi for pyrophosphate; Pi for inorganic phosphate. SAMS produce SAM from methionine. SAM is the methyl donor for most transmethylation reactions. The product, SAH, inhibits transmethylation by competing with SAM for methyltransferases (MTases). SAH is converted to homocysteine (Hcy) and adenosine by S-adenosyl homocysteine hydrolase (SAHH). Phosphorylation of adenosine by adenosine kinase (ADK) is critical because the SAHH-catalyzed reaction is reversible and the equilibrium lies in the direction of SAH synthesis. Geminivirus AC2/AL2 and C2/L2 proteins inactivate ADK and has also been shown to stabilize SAM decarboxylase (SAMDC), which causes decarboxylated SAM (dcSAM) levels to rise. TYLCCNV betasatellite encoded βC1 protein directly antagonizes the methyl cycle by inhibiting SAHH. Data in this report indicates that the CLCuMuV C4 protein inhibits SAMS activity by interacting directly.

Based on previous research and our work, we propose a working model for C4-SAMS interaction in the regulation of gene silencing. Host methylation modifies the geminivirus genome and protects viral RNA-targeting siRNAs from degradation, promoting TGS and PTGS defense against geminiviruses. It seems that methylation interference via methyl cycle inhibition is a common approach against geminiviruses to counter host methylation dependent silencing defense (Fig 7). In this model, CLCuMuV-encoded C4 directly targets the core methyl cycle enzyme SAMS and inhibits SAMS activity to generate SAM by the adenylation of methionine. SAM is the methyl donor for most transmethylation reactions. CLCuMuV C4-mediated reduction of SAM levels further decrease viral DNA methylation, promote the stability of geminivirus RNA and enhance virus infection. In this scenario, we cannot exclude the possible involvement of polyamines and viral protein methylation in CLCuMuV infection, because SAM is also the methyl donor for various methylation. Recently, a viral protein was shown to promote ethylene production for the benefit of virus infection by enhancing host SAMS1 activity [43]. It will need further investigation whether CLCuMuV C4 contributes to viral infection by inhibiting the biosynthesis of polyamines and ethylene or viral proteins.

## Materials and methods

### Plant materials and agroinfiltration

Wild-type *N*. *benthamiana*, *N*. *benthamiana* line 16c containing a GFP transgene [57] (provided by xueping Zhou lab) and 16c-TGS a transcriptionally silenced Green Fluorescent Protein (GFP) transgenic line, was generated as described [21]. *N*. *benthamiana* plants were grown at 25 °C under a 16-h-light/8-h-dark cycle. Four to five weeks old plants were used in experiments. All experiments were conducted at least three times, with at least 7 plants per construct each time.

### Plasmid constructs

The infectious clones of CLCuMuV and its betasatellite CLCuMuB, CLCuMuB-based VIGS vector βM2 and its derivative βM2-GFPF (βM2 containing GFP fragment) were used as described previously[45]. The βM2-LUC was generated by replacing GFP sequence of βM2-GFPF with 345 bp of luciferase fragment LUC sequence. CLCuMuV-C4^R13A^ was generated by replacing CLCuMuV *C4* with *C4*^*R13A*^ by overlapping PCR. The infectious clones of TYLCCNV and its betasatellite Y10β were previously described [58]. Y10mβ-LUC and Y10mβ-C4 were generated respectively by replacing the *βC1* of Y10β with 345bp of (LUC) and CLCuMuV *C4* sequences.

DNA fragment of NbSAMS2, C4 and C4^R13A^ were PCR amplified respectively, and then cloned into NdeI-XhoI-digested pGEX4T-1 vector to express GST-tagged fusion proteins GST-NbSAMS2, GST-C4 and GST-C4^R13A^ in *E*. *coli*. Full-length C4 and C4^R13A^ was individually cloned into BamHI-XhoI-digested pET28a to express C4-His and C4 ^R13A^-His in *E*. *coli*.

For generating T-DNA based fusion protein expression constructs by ligation-independent cloning (LIC)-based method, various LIC-based expression vectors were generated as below: pcLUC-LIC was described, and generated by cloning LIC cassette containing *ccdB* gene flanking LIC adaptors with *Apa*I site (PCR amplified using pYL436 (GenBank Accession: AY737283) as a template) into pCAMBIA-cLUC [41]. pcLUC-LIC is used to generate cLUC fusion protein constructs. *NbSAMS1*, *NbSAMS2* and *NbSAMS3* ORFs were PCR amplified, and then cloned into pcLUC-LIC to cLUC-NbSAMS1, cLUC-NbSAMS2 and cLUC-NbSAMS3 expression constructs by LIC method as described.

LIC-pnLUC was generated by cloning LIC cassette containing *ccdB* gene flanking LIC adaptors with *Apa*I site (PCR amplified using pYL436 as a template) into pCAMBIA-nLUC [41]. C4 and C4^R13A^ were PCR amplified, and then cloned into LIC-pnLUC to C4-nLUC, C4^R13A^-nLUC expression constructs by LIC method as described [59].

LIC-pGFP, LIC-pnYFP, pcYFP-LIC and LIC-Pha were generated by replacing cLUC sequence of pcLUC-LIC with DNA fragments (without stop codon) of GFP, nYFP, cYFP and 3×HA sequence respectively. DNA fragments of C4, C4^R13A^, an N-terminal part of C4 (N50, amino acids 1–50), M-terminal part of C4 (M60, amino acids 21–80) and C-terminal part of C4 (C50, amino acids 51–100) were PCR amplified respectively, and cloned into LIC-pGFP vector to generate C4-GFP, C4^R13A^-GFP, C4-N50-GFP, C4-M60-GFP, C4-C50-GFP expression constructs. DNA fragments of C4 and C4^R13A^ were cloned into LIC-pnYFP to C4-nYFP, C4^R13A^-nYFP expression constructs. cLUC DNA fragment was cloned into LIC-pHA to generate cLUC-HA expression construct.

NbSAMS2 ORF was PCR amplified, and cloned into pcYFP-LIC and pHA-LIC respectively to generate cYFP-NbSAMS2 and HA-NbSAMS2 expression constructs. DNA fragments of C4-HA, C4^R13A^-HA and cLUC-HA were PCR amplified, and then cloned into PVX-LIC vector [60] by LIC method to generate PVX-based expression constructs PVX-C4-HA, PVX-C4^R13A^-HA and PVX-cLUC-HA.

Before LIC cloning, all LIC-based expression vectors except PVX-LIC were digested with *Apa*I, while PVX-LIC vector was digested with SmaI. LIC cloning was performed as described. For LIC cloning, digested LIC-based expression vector was further treated with T4 DNA polymerase in the presence of dTTP (0.5 mM) and DTT (1 mM) for 30 min at 37 °C to produce the 14 nt sticky 5’-end. After the polymerase was inactivated at 75 °C for 20 min, the sticky-ended LIC-based expression vector was purified by phenol extraction and ethanol precipitation. PCR product was treated with T4 DNA polymerase in the presence of dATP (0.5 mM) and DTT (1 mM) for 30 min at 37 °C, followed by a 75 °C inactivation step and purified by ethanol precipitation. The equal volumes of LIC-based expression vector and PCR product treated with T4 DNA polymerase were mixed and incubated at 37 °C for 30 min, and then transformed into *E*. *coli*. strain DH5α [60]. The resulting constructs were verified by sequencing.

Primers sequences and information used for plasmid construction in this study are listed in S1 Table.

### Mass spectrometry analysis

Total proteins were extracted as described previously [61] from *N*. *benthamiana* leaves infected with C4-GFP or GFP constructs respectively. The total protein extracts were incubated with GFP-Trap_A beads (ChromoTek). After washing, the purified GFP-tagged C4 proteins were denatured at 98 °C and separated by SDS-PAGE gel (12%) and visualized by silver staining. Protein bands were excised and in-gel digested with trypsin (Promega) and the peptides were extracted twice with 1% (v/v) trifluoroacetic acid in 50% (v/v) acetonitrile aqueous solution for 30 min. All the peptides were subjected to LC-MS/MS analysis as described previously [61].

### Protein analyses and co-IP

For protein analyses, proteins were transiently expressed by agroinfiltration in *N*. *benthamiana* leaves and harvested at 2 or 3 dpi. Total proteins were extracted with a ratio of 1:1 of 2 × Laemmli buffer. After boiling for 10 min, protein extracts were separated by SDS-PAGE for immunoblot analysis using indicated antibodies. HA-tagged SAMS (HA-NbSAMS2) was transiently coexpressed with GFP or GFP-tagged C4 or C4^R13A^- GFP (C4-GFP), in *N*. *benthamiana*. Leaf tissues were then collected at 60 hours of post inoculation (hpi). Co-IP experiments was performed as described previously[62]. Total protein extracts were immunoprecipitated using anti-GFP antibody coupled to agarose beads, and the resulting precipitates were analyzed by immunoblot using anti-HA antibodies.

### GST pull-down assays

For GST pull-down assay, GST-NbSAMS2 and C4-6×His or C4^R13A^-6×His fusion proteins were produced in BL21 (DE3) cells (Stratagene). GST- NbSAMS2 was purified using glutathione-Sepharose beads (GE Healthcare) according to the manufacturer’ s instructions. The GST pull-down assays were performed as described previously[63].

### BiFC assay

For BiFC assay, proteins were transiently expressed by agroinfiltration in *N*. *benthamiana* leaves. The experimental group and corresponding control group were inoculated in a same leaf to reduce the difference of expression condition. The leaves were detached 60 hpi, and confocal imaging was acquired by Zeiss LSM 710 laser scanning microscope (Carl-Zeiss).

### LCI assays

LCI assays were performed as described [64]. All combinations tested were agroinfiltrated into leaves of *N*. *benthamiana*. The leaves were detached 60 hpi, sprayed with 1 mM luciferin, and observed under a low-light cooled CCD imaging apparatus (iXon; Andor Technology). The photos were taken 5 min after exposure.

### SAMS enzymatic activity assays

Direct assays of SAMS activity were performed according to the scheme presented in Fig 2A, using SAM production by SAMS as a measure of SAMS-catalyzed MET that yields SAM and free phosphor. The fusion proteins GST-C4, GST-C4R^13A^ and GST-NbSAMS2 were produced in BL21(DE3) codon plus RIL cells and purified using Glutathione Sepharose 4B (GE, USA). Target proteins were collected by elution buffer (300 mM NaCl, 50 mM Tric-HCl, 10 mM Reduced Glutathione) at 4°C. Mixtures containing, in a total volume of 15 μl, 10 ng SAMS, and various amounts of C4 were pre-incubated at 30 °C for 20 min. Mixtures were then added to reactions containing (final concentrations) 50 mM Tris-HCl, pH 7.6, 5 μCi 35S-Met (3000 Ci/mmol), 50 mM ATP and 10 mM MgCl_2_. Reactions were incubated at 30 °C for 20 min, when SAMS activity was terminated by addition of 1 μl of 1 M EDTA. SAM production was analyzed by thin layer chromatography on poly ethylene iminecellulose plates developed with 1M acetic acid [65]. After chromatography, radioactive signals on plates were quantitated using a phosphor imager (Bio-Rad Molecular Imager FX).

### GFP imaging and virus inoculation

GFP recovery assays were performed as described [9,21]. Briefly, 16c-TGS plants were agroinoculated with PVX vectors. After the primary harvest, plants were allowed to continue growing under the same conditions and symptom development was observed in new secondary shoots after additional 2–3 weeks. GFP expression was evaluated under long-wavelength UV light and photographed with a Nikon 5000 digital camera (Tokyo, Japan).

### DNA isolation, bisulfite sequencing

Genomic DNA was extracted from plant leaf samples using the DNeasy Plant Mini kit (Qiagen, Valencia, CA). To improve the efficiency of bisulfite treatment, DNA (1 mg) was digested with a restriction enzyme that cuts outside the region of interest to decrease the size of DNA, followed by overnight treatment with proteinase K. Bisulfite modification was carried out using the EZ DNA Methylation Gold kit (Zymo Research, Irvine, CA) in a PCR machine. Bisulfite-modified DNA was purified using a Zymo-Spin IC column and dissolved in 10 μl of Elution Buffer according to the manufacturer’s instructions. PCR amplification was then carried out using ZymoTaq and products were cloned into a pGEM-T easy vector (Promega). Individual clones were sequenced. Primers were designed against templates and are listed in S1 Table. The statistical analysis was performed using the OriginPro 8 program. The bars denote the SE of the means, asterisks are representing significantly different from each group (one-way analysis of variance, *p<0.05).

### Real-time RT-PCR

Total RNA was extracted from apical developing leaves using the Trizol reagent (TIANGEN, China) and treated with RNase-free DNase I (Sigma-Aldrich). First strand cDNA was synthesized using 2–5 μg of total RNA with oligo-d(T) primer and M-MLV reverse transcriptase (TIANGEN, China). Real time RT-PCR was performed using SYBR Green-based real-time PCR, a 10 μL reaction mixture containing 5 μl Power SYBR Green PCR Master Mix (2×) (Life, USA), 0.1 μL of each 20 μM primer and 0.3 μL, 60 ng/μL templet were chosen to amplify target sequences. PCR amplification procedures: the first step: 95 °C to 5 minutes. The second step: 95 °C for 5 seconds, 60 °C for 30 seconds, 40 cycles. Third step: from the beginning of 65 degrees, every 5 seconds to increase the temperature of 0.5 degrees, until the end of the reaction at 95°C. *eIF4a* was used as internal control for *N*. *benthamiana* for normalization. The values were calculated using the comparative normalized Ct method [66] and all the experiments were repeated at least three times. Data were analyzed and plotted with Origin 8.1.

### Geminivirus based VIGS and real-time PCR

Geminivirus-based VIGS assay and viral replication determination were performed as described previously[45]. Total DNA was extracted from apical developing leaves using the DNAsecure Plant Kit (TIAN-GEN, China). A single copy of CLCuMuV genome was amplified by PCR and then was ligased into pMD19-T (TaKaRa, Japan) to generate a CLCuMuV-positive plasmid. A 10-fold serial dilution of the plasmid DNA from 2×10^8^ to 200 copy was prepared and used as the standard. A CLCuMuV-specific primer set (qCLCuMuV V1-F and qCLCuMuV V1-R) was used to amplify a 198-bp amplicon. Because the standard curves generated were linear in the whole range tested with a coefficient of regression R^2^:0.99 and calculated slope around -3.5 for SYBR Green assay. The copy number of viral DNA can be calculated via Ct value of each sample and the standard curve.

To obtain the ratio of viral DNA: plant genome DNA, Plant genome DNA can also be calculated via internal reference method. The genome DNA of healthy *N*. *benthamiana* was extracted and a 2-fold serial dilution of the genome DNA from 145ng to 1.13ng was prepared and used as the standard. An eIF4a-specific primer set (qeIF4a-F and qeIF4a-R) was used to amplify a 60-bp amplicon. The plant genome DNA can be calculated via Ct value of each sample and the standard curve.

### Southern blot analysis

Total DNA was extracted from apical developing leaves using the DNAsecure Plant Kit (TIAN- GEN, China). Total DNAs (100 ng DNA) separated electrophoretically in 1% agarose gels containing chloroquine (20 μg/ml), and analyzed by Southern blot hybridization with biotin-labeled probes specific for CLCuMuV. The DNA agarose gel was stained with ethidium bromide as a loading control. After denaturation and neutralization, total DNA was transferred to Hybond N+nylon membranes (GE Healthcare, Pittsburgh, PA). Membranes were hybridized at 55°C to specific probes.

### siRNA blot analysis

To analyze the production siRNAs, low-molecular-mass RNAs were enriched from total RNA as described previously [67]. The enriched small RNAs (15 mg) were fractionated on a 15% denaturing polyacrylamide–7 M urea gel in 0.5 × Tris–borate EDTA (TBE) buffer. The RNA was transferred to Hybond N^+^ membranes (GE Healthcare) by electroblotting in 0.5 × TBE at 400 mA for 1 h. The transferred RNAs was UV crosslinked to the membrane 4 times at 1200 mJ in a UV Stratalinker 1800 (Stratagene, La Jolla, CA). Membranes were stored at 4 °C until probing. One DNA oligonucleotides complementary to *N*. *benthamiana* U6 RNA and a mixture of oligonucleotides corresponding to G, F and P regions of GFP mRNA sequences were synthesized and used as probes for siRNA hybridization. The oligos were end-labelled with [γ-^32^P] ATP in 50 mL reactions containing 1 mM DNA oligo and 7 U T4 polynucleotide kinase. Hybridizations were performed overnight at 42 °C and the membranes were subsequently washed three times (10 min each) at 40 °C with 1 × SSC (0.15 M NaCl and 0.015 M sodium citrate) supplemented with 0.1% SDS. Hybridization signals were detected as described above for Northern blot analysis.

### Protoplast isolation

Protoplast isolation assay was performed as described previously [68]. We isolated protoplasts from the leaves of *N*. *benthamiana* plants, and then transfected them with CLCuMuVΔRep and expression construct of either Rep or Rep^E65G^.

### Accession number

Sequence data from this article can be found in the GenBank data libraries under accession numbers: CLCuMuV (EF465535); CLCuMuV isolate Okra (GU574208.1); CLCuMuB (EF465536); TYLCCNV (AJ319675); TYLCCNB (AJ781300.1); *NbSAMS1* (KX452091); *NbSAMS2* (KX452092); *NbSAMS3* (KX452093); *NbTnt1* (AJ228076.1); *eIF4a* (KX247369); GFP (U87973).

## Supporting information

S1 Fig Silver-stained SDS-PAGE gel and LC-MS/MS results. **(A)** Silver-stained SDS-PAGE gel. **(B)** Representative tandem mass spectrum (MS/MS spectrum) for a peptide from NbSAMS2 protein (peptide sequence: FVIGGPHGDAGLTGR). The + and 2+ in the diagram indicate the valence state of ions is monovalent or divalent.
(TIF)

S2 Fig Alignment of SAMS homologs from *N*. *benthamiana*. The alignment was generated using Clustal W2. Gray backgrounds represent residues that are conserved in 100% of the sequences at the corresponding positions. Lowercased letters under each block indicate residues that are consensus in all aligned sequences. Numbers at the right indicate the positions of amino acid residues.
(TIF)

S3 Fig C4 interacts with NbSAMS homologs and N-terminal part of C4 is responsible for its binding to NbSAMS2. **(A)** LCI assays showed the interaction of NbSAMS homologs with CLCuMuV C4 in plants. Image shown is luminescence of *N*. *benthamiana* leaf that was agro infiltrated with C4-nLUC or C4^R13A^-nLUC with the cLUC-tagged NbSAMS homologs. NbATG3-nLUC+cLUC-NbGAPC3 was served as positive control while cLUC-HA+ C4-nLUC was negative control. The experiments were repeated three times with similar results. nLUC represents N-terminal and cLUC represents C-terminal fragment of the firefly luciferase. **(B)** Schematic representation of the truncated mutants of C4. **(C)** Co-IP assays showed that only N-terminal part of C4 interacted with NbSAMS. Total protein extracts were immunoprecipitated with anti-GFP beads, and then separated by SDS-PAGE for immunoblotting (IB) using anti-GFP or anti-HA antibodies. **(D)** Localized of C4 and C4^R13A^. C4-YFP and C4^R13A^-YFP transiently expressed in N.benthamiana leaves respectively, and examined by confocal laser scanning microscopy at 60 hpi. Yellow color represents C4-YFP or C4^R13A^-YFP.
(TIF)

S4 Fig TYLCCNV is able to support βM2 replication but cannot reverse TGS of a GFP transgene when co-inoculated with βM2. **(A)** BiFC assay controls. Cells were photographed 60 hpi using confocal laser scanning microscope. Bar scale represents 50 μm. **(B)** *N*. *benthamiana* 16c-TGS plants were inoculated with TYLCCNV or TYLCCNV plus βM2-nLUC, and photographed under UV light at 21 dpi. nLUC represents N-terminal fragment of the firefly luciferase. **(C)** PCR was performed to confirm the presence of TYLCCNV or βM2-nLUC using TYLCCNV *V1* gene-specific and βM2 IR region-specific primers.
(TIF)

S5 Fig The effect of *NbSAMS2* silencing using TYLCCNV as a helper virus on mRNA levels of various *SAMS* genes and TYLCCNV DNA accumulation. **(A–C)**. Transgenic *N*. *benthamiana* 16-TGS plants were co-inoculated with TYLCCNV and βM2 vector containing DNA fragment of NbSAMS2 or nLUC. Relative mRNA levels of *NbSAMS2* **(A)**, *NbSAMS1* **(B)** and *NbSAMS3* **(C)** were analyzed by real-time RT-PCR using gene-specific primers. *eIF4α* was used as an internal control. Values represent means ± SE from three independent experiments. (*p<0.05). **(D)** Viral DNA accumulation. Real-time PCR analysis of *V1* gene from TYLCCNV was used to determine viral DNA level. Values represent means ± SE from three independent experiments. (*p<0.05).
(TIF)

S6 Fig The phenotype of *NbSAMS2* silenced plants using TRV-based VIGS vector. The phenotype of *NbSAMS2* silenced *N*. *benthamiana*, photographed at 25 dpi. SAMS2-silenced plants showed a severe phenotype.
(TIF)

S7 Fig The efficiency of *NbSAMS2* silencing using TRV-based VIGS vector. **(A–C)** Relative mRNA levels of *NbSAMS2* **(A)**, *NbSAMS1* **(B)** and *NbSAMS3* **(C)** were analyzed by real-time RT-PCR using gene-specific primers. *eIF4α* was used as an internal control. Values represent means ± SE from three independent experiments. (*p<0.05).
(TIF)

S8 Fig Relative mRNA levels of NbSAMS1 and NbSAMS3 in NbSAMS2 silenced plants using CLCuMuV as a helper virus. **(A-C)** mRNA levels of NbSAMS1, NbSAMS2 and NbSAMS3 in NbSAMS2 silenced plants respectively. Silencing was performed by using CLCuMuV as a helper virus. Real-time RT-PCR was performed using gene-specific primers. *eIF4α* was used as an internal control. Values represent means ± SE from three independent experiments. (*p<0.05).
(TIF)

S9 Fig TYLCCNV-based C4 expression restored TGS suppression of GFP. **(A)** TYLCCNV-based C4 expression reversed expression of a GFP transgene. *N*. *benthamiana* 16c-TGS plants were inoculated with TYLCCNV, TYLCCNV+ Y10mβ-mGFP, TYLCCNV+ Y10mβ-C4 or TYLCCNV+Y10β (as positive control). Plants were photographed under UV light at 14 dpi. **(B)** Western blot assay of GFP accumulation in inoculated plants. GFP protein level was assessed by anti-GFP antibody. Ponceau Red stained of Rubisco was used as a protein loading control. **(C)** The expression of C4 increased TYLCCNV DNA accumulation. Real-time PCR analysis of *V1* gene from TYLCCNV was used to determine viral DNA level. Values represent means ± SE from three independent experiments.
(TIF)

S10 Fig Overexpression of C4 suppresses PTGS pathway. **(A)** GFP fluorescence in leaves of *N*. *benthamiana* plants transiently expressing 35S-GFP together with indicated suppressors. **(B)** Real-time RT-PCR showed relative GFP mRNA levels of leaves of 16-TGS plants inoculated as indicated. Values represent means ± SE from three independent experiments. (*p<0.05). **(C)** Western blot assay of GFP accumulation in inoculated plants shown in **(A)**. GFP protein level was assessed by anti-GFP antibody. Ponceau Red Stained Rubisco was used as a protein loading control. **(D)** Small RNA gel blot analyses of GFP silencing in agroinfiltrated leaf samples. [γ- ^32^P] CTP-labeled GFP or U6 oligonucleotides were used as probes in the small RNA blots. The sizes of the 21-, 22- and 24-nt RNAs are indicated to the right of the small RNA panel.
(TIF)

S1 Table Primers sequences used in vector construction and PCR analysis. (DOCX)
