# Supplementary figures and images for: Cotton Leaf Curl Multan virus C4 protein suppresses both transcriptional and post-transcriptional gene silencing by interacting with SAM synthetase
Source: PLoS Pathog. 2018 Aug 29;14(8):e1007282. doi: 10.1371/journal.ppat.1007282 (PMC6133388; doi:10.1371/journal.ppat.1007282)

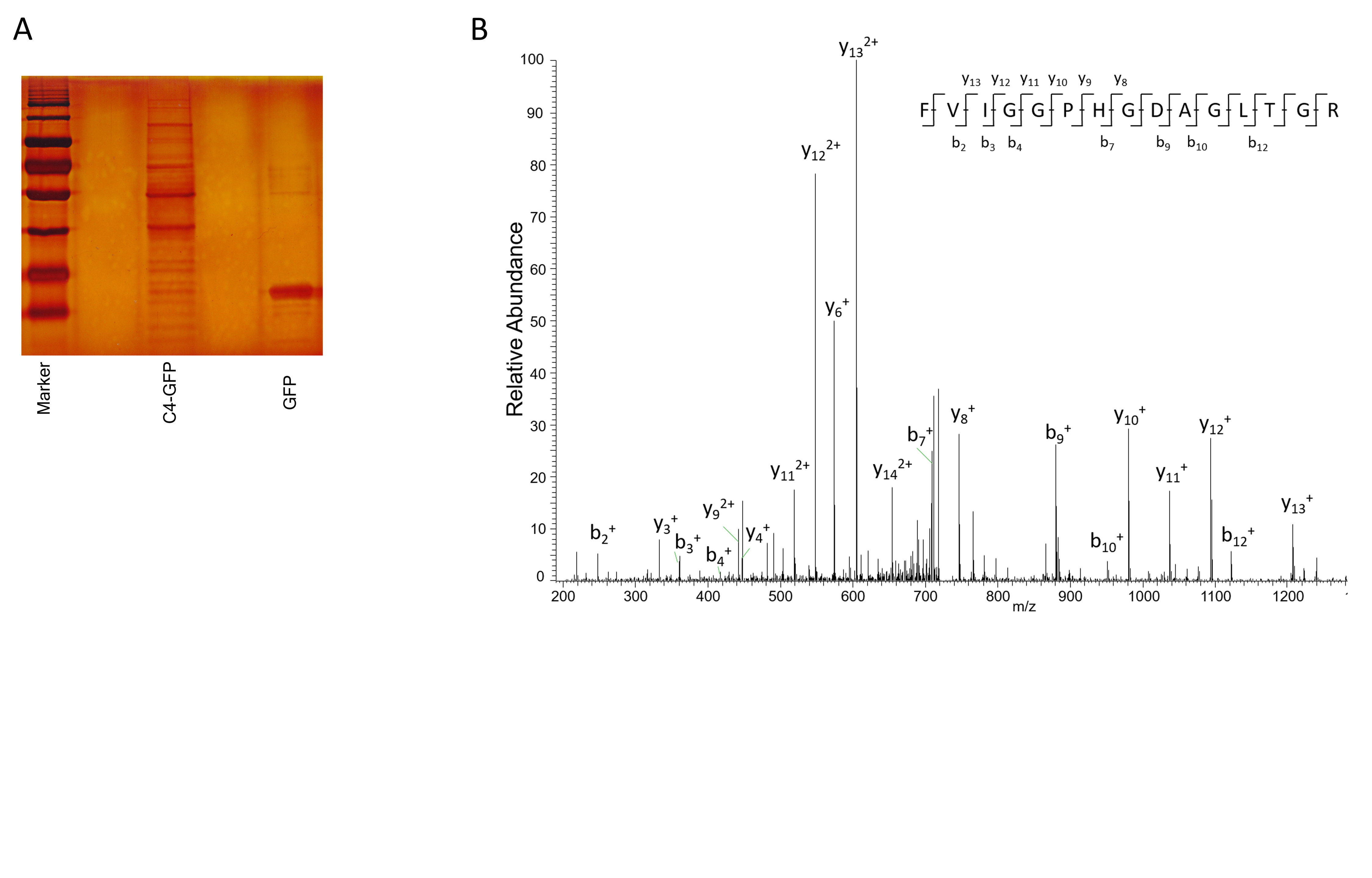

Supplement: S1 Fig — (A) Silver-stained SDS-PAGE gel. (B) Representative tandem mass spectrum (MS/MS spectrum) for a peptide from NbSAMS2 protein (peptide sequence: FVIGGPHGDAGLTGR). The + and 2+ in the diagram indicate the valence state of ions is monovalent or divalent. (TIF) [file ppat.1007282.s001.tif]

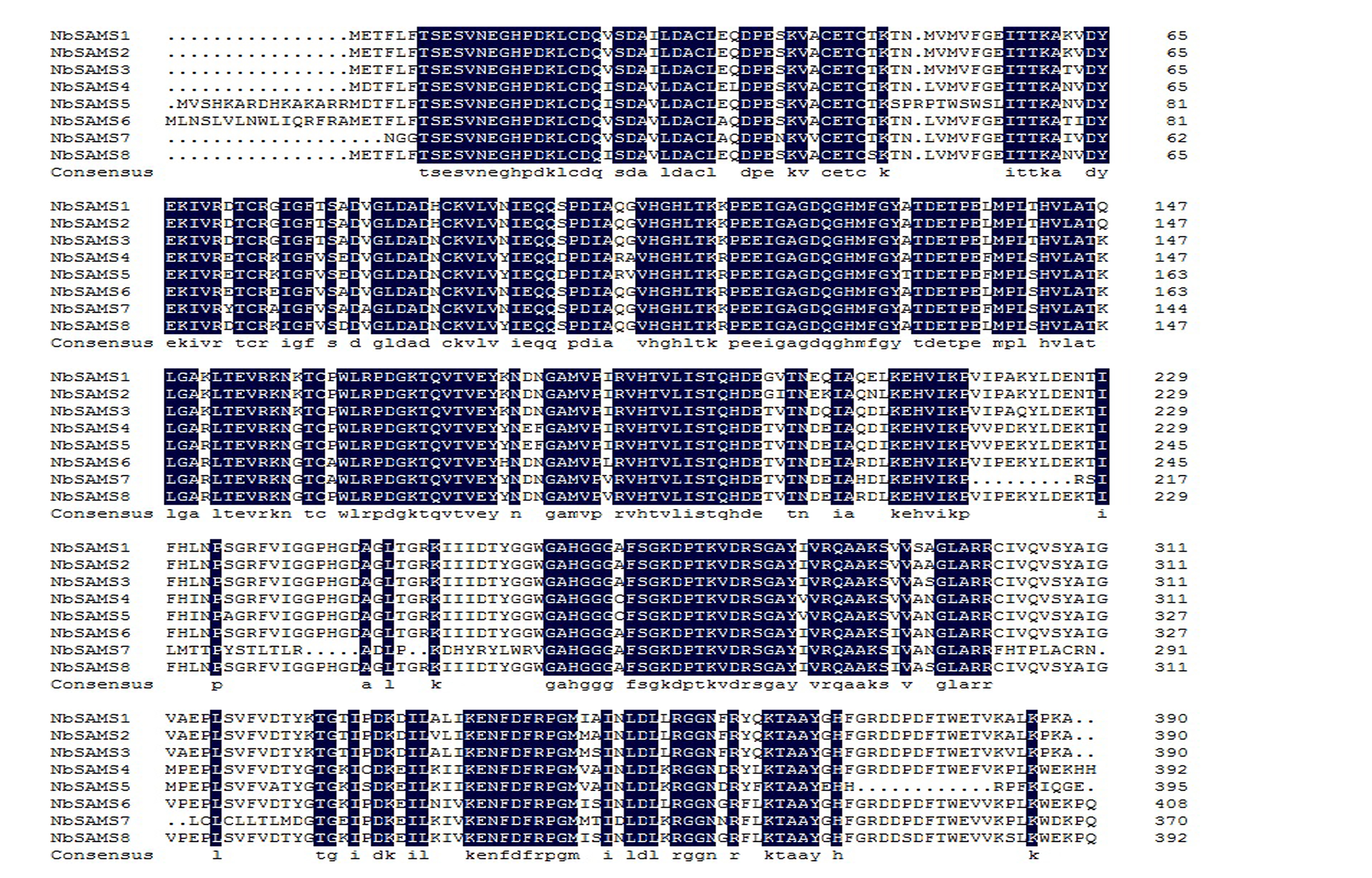

Supplement: S2 Fig — The alignment was generated using Clustal W2. Gray backgrounds represent residues that are conserved in 100% of the sequences at the corresponding positions. Lowercased letters under each block indicate residues that are consensus in all aligned sequences. Numbers at the right indicate the positions of amino acid residues. (TIF) [file ppat.1007282.s002.tif]

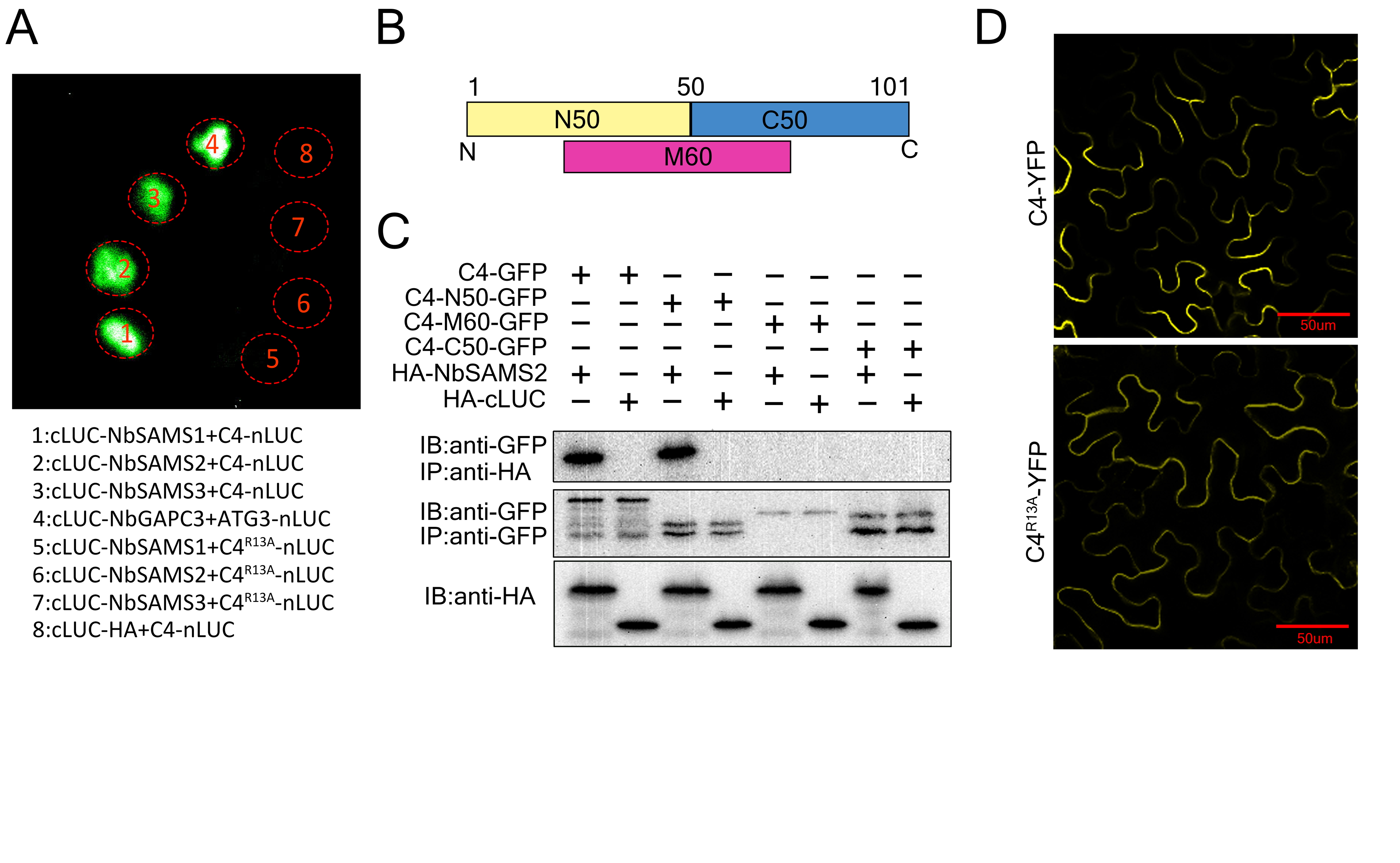

Supplement: S3 Fig — (A) LCI assays showed the interaction of NbSAMS homologs with CLCuMuV C4 in plants. Image shown is luminescence of N. benthamiana leaf that was agro infiltrated with C4-nLUC or C4R13A-nLUC with the cLUC-tagged NbSAMS homologs. NbATG3-nLUC+cLUC-NbGAPC3 was served as positive control while cLUC-HA+ C4-nLUC was negative control. The experiments were repeated three times with similar results. nLUC represents N-terminal and cLUC represents C-terminal fragment of the firefly luciferase. (B) Schematic representation of the truncated mutants of C4. (C) Co-IP assays showed that only N-terminal part of C4 interacted with NbSAMS. Total protein extracts were immunoprecipitated with anti-GFP beads, and then separated by SDS-PAGE for immunoblotting (IB) using anti-GFP or anti-HA antibodies. (D) Localized of C4 and C4R13A. C4-YFP and C4R13A-YFP transiently expressed in N.benthamiana leaves respectively, and examined by confocal laser scanning microscopy at 60 hpi. Yellow color represents C4-YFP or C4R13A-YFP. (TIF) [file ppat.1007282.s003.tif]

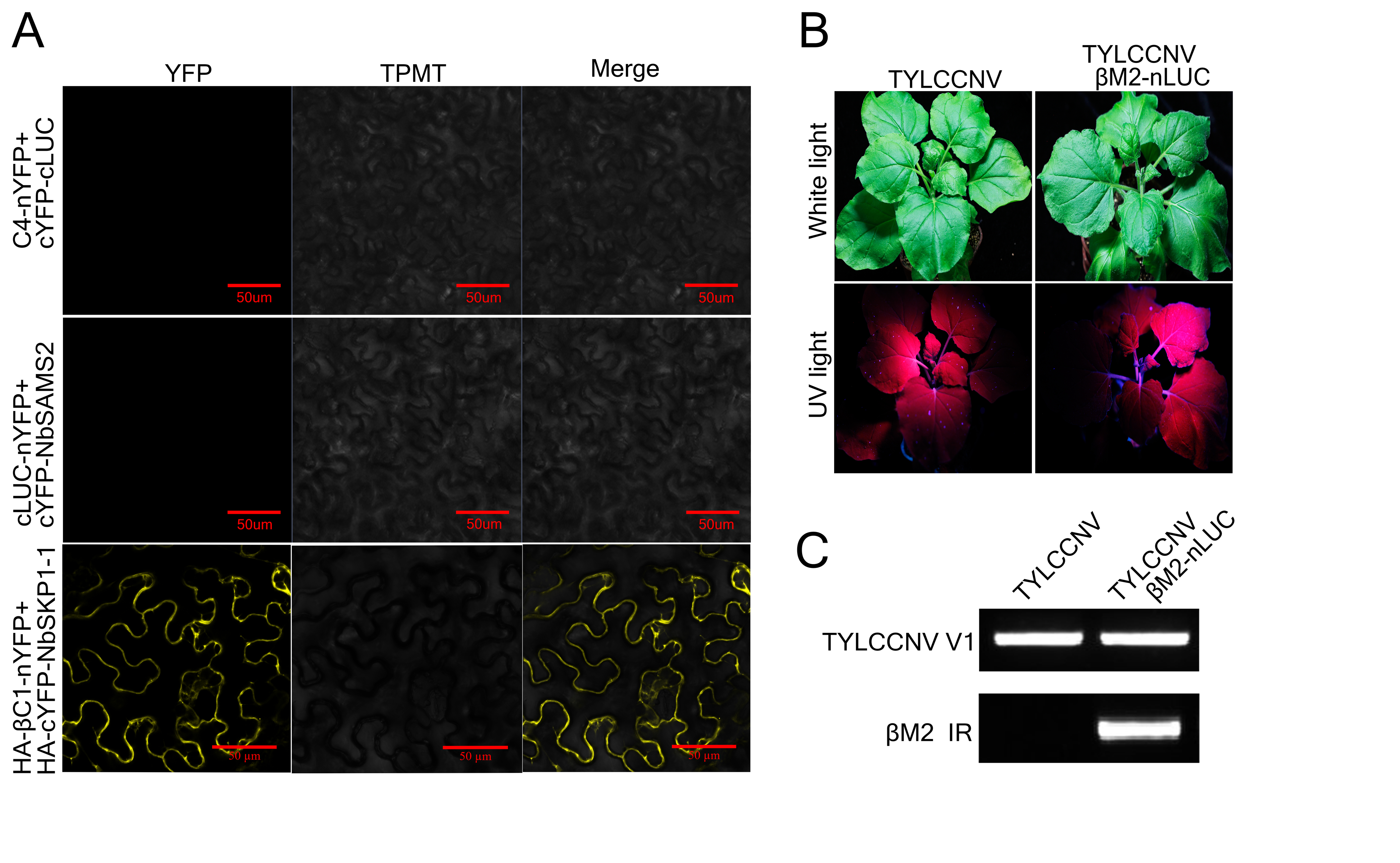

Supplement: S4 Fig — (A) BiFC assay controls. Cells were photographed 60 hpi using confocal laser scanning microscope. Bar scale represents 50 μm. (B) N. benthamiana 16c-TGS plants were inoculated with TYLCCNV or TYLCCNV plus βM2-nLUC, and photographed under UV light at 21 dpi. nLUC represents N-terminal fragment of the firefly luciferase. (C) PCR was performed to confirm the presence of TYLCCNV or βM2-nLUC using TYLCCNV V1 gene-specific and βM2 IR region-specific primers. (TIF) [file ppat.1007282.s004.tif]

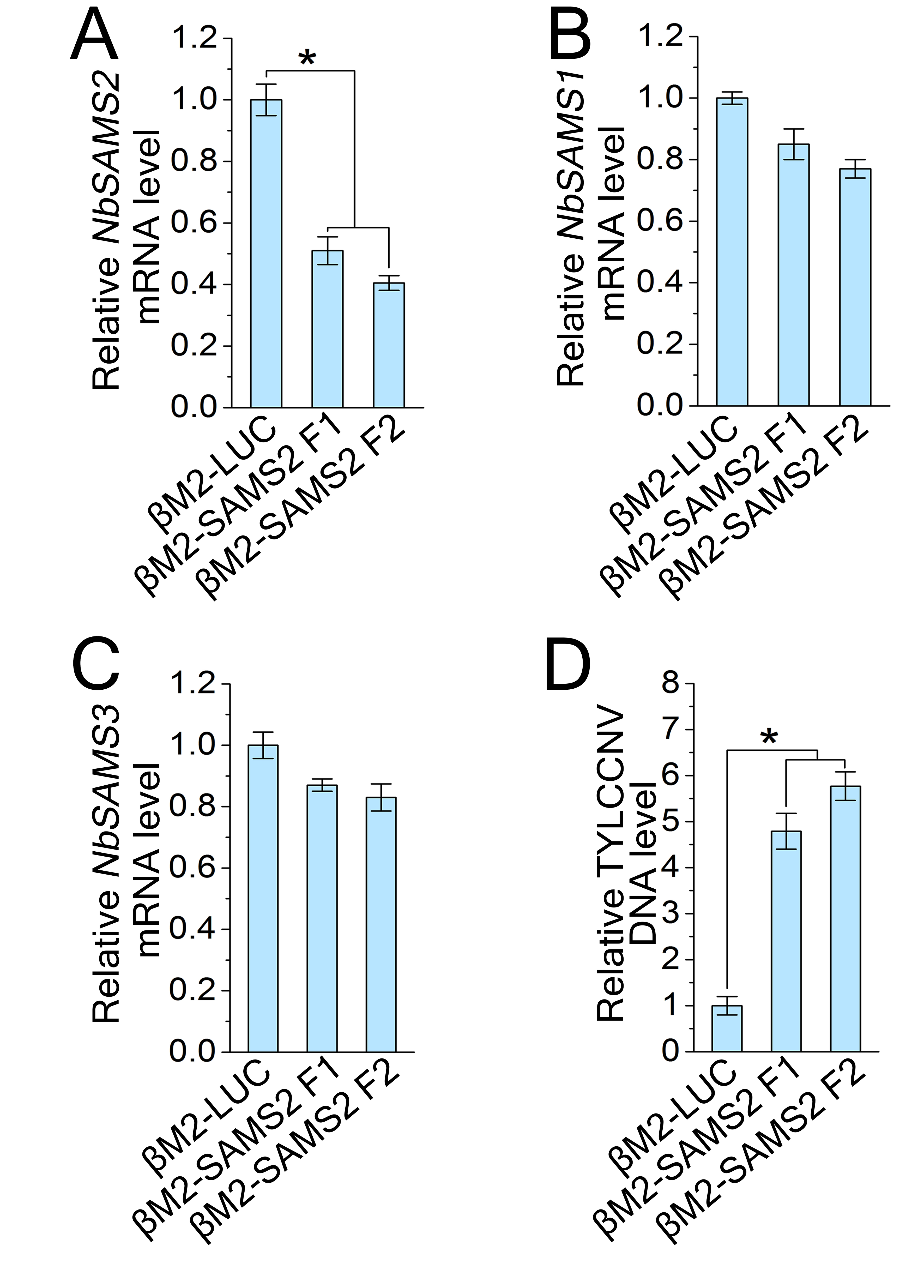

Supplement: S5 Fig — (A–C). Transgenic N. benthamiana 16-TGS plants were co-inoculated with TYLCCNV and βM2 vector containing DNA fragment of NbSAMS2 or nLUC. Relative mRNA levels of NbSAMS2 (A), NbSAMS1 (B) and NbSAMS3 (C) were analyzed by real-time RT-PCR using gene-specific primers. eIF4α was used as an internal control. Values represent means ± SE from three independent experiments. (*p<0.05). (D) Viral DNA accumulation. Real-time PCR analysis of V1 gene from TYLCCNV was used to determine viral DNA level. Values represent means ± SE from three independent experiments. (*p<0.05). (TIF) [file ppat.1007282.s005.tif]

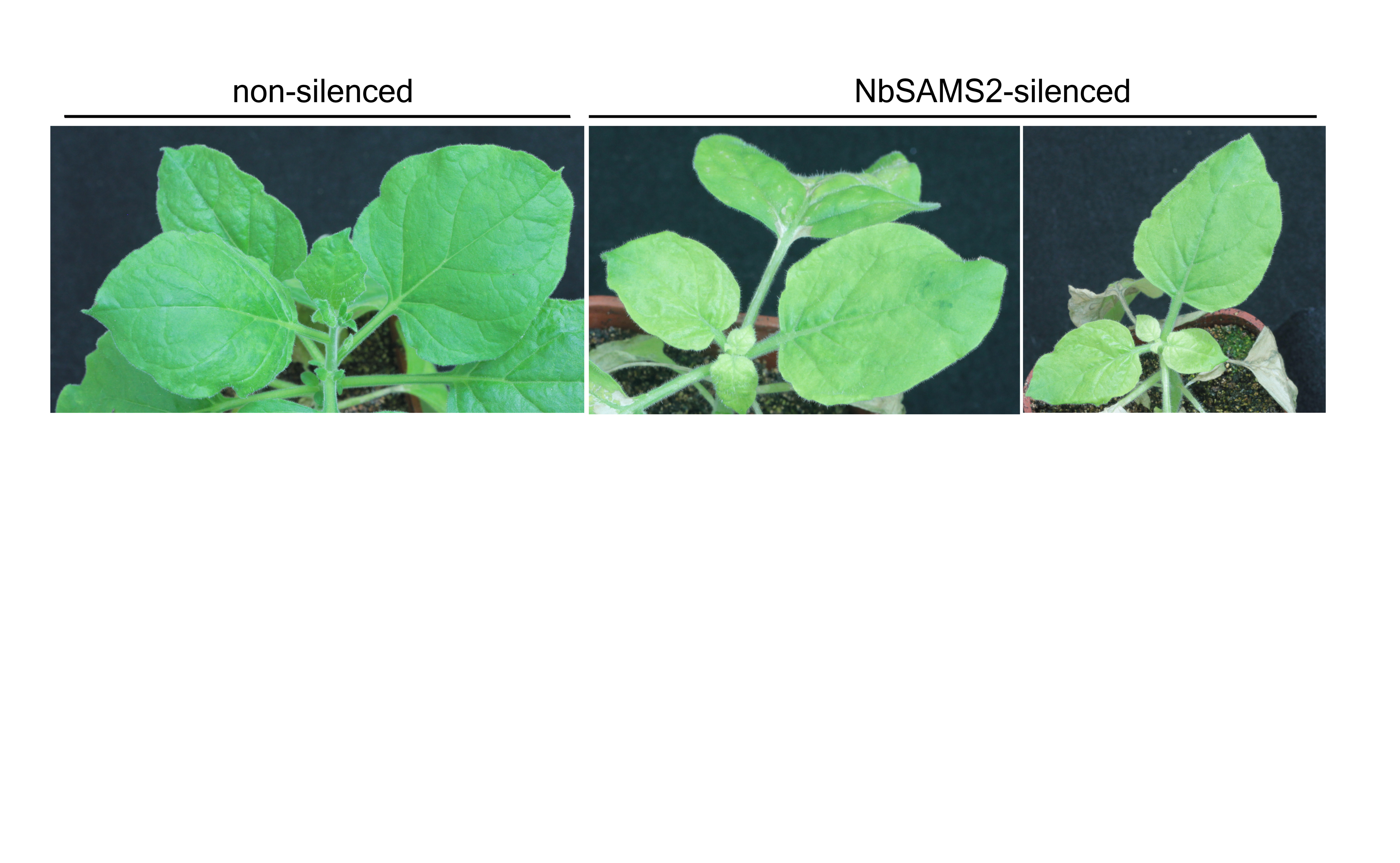

Supplement: S6 Fig — The phenotype of NbSAMS2 silenced N. benthamiana, photographed at 25 dpi. SAMS2-silenced plants showed a severe phenotype. (TIF) [file ppat.1007282.s006.tif]

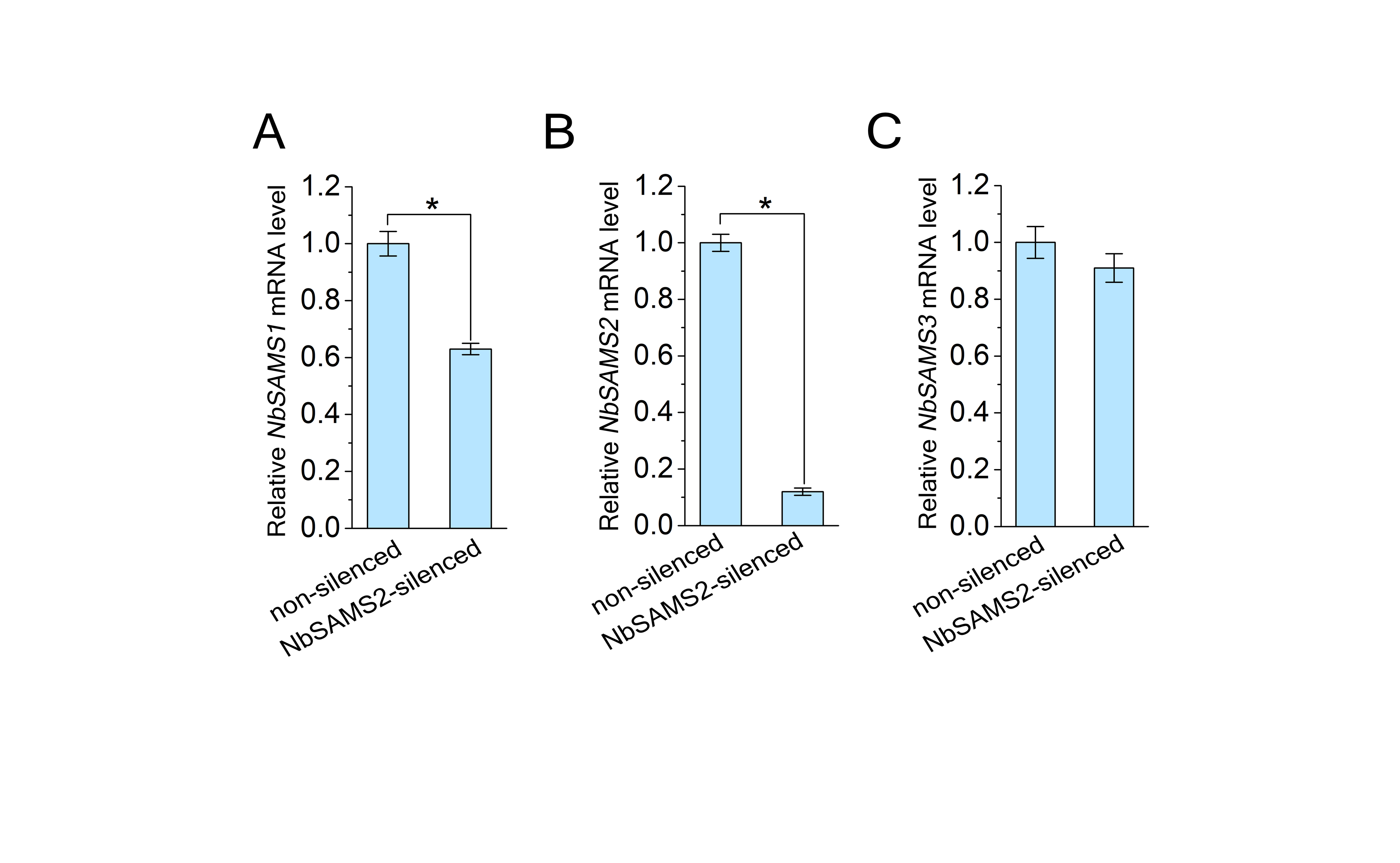

Supplement: S7 Fig — (A–C) Relative mRNA levels of NbSAMS2 (A), NbSAMS1 (B) and NbSAMS3 (C) were analyzed by real-time RT-PCR using gene-specific primers. eIF4α was used as an internal control. Values represent means ± SE from three independent experiments. (*p<0.05). (TIF) [file ppat.1007282.s007.tif]

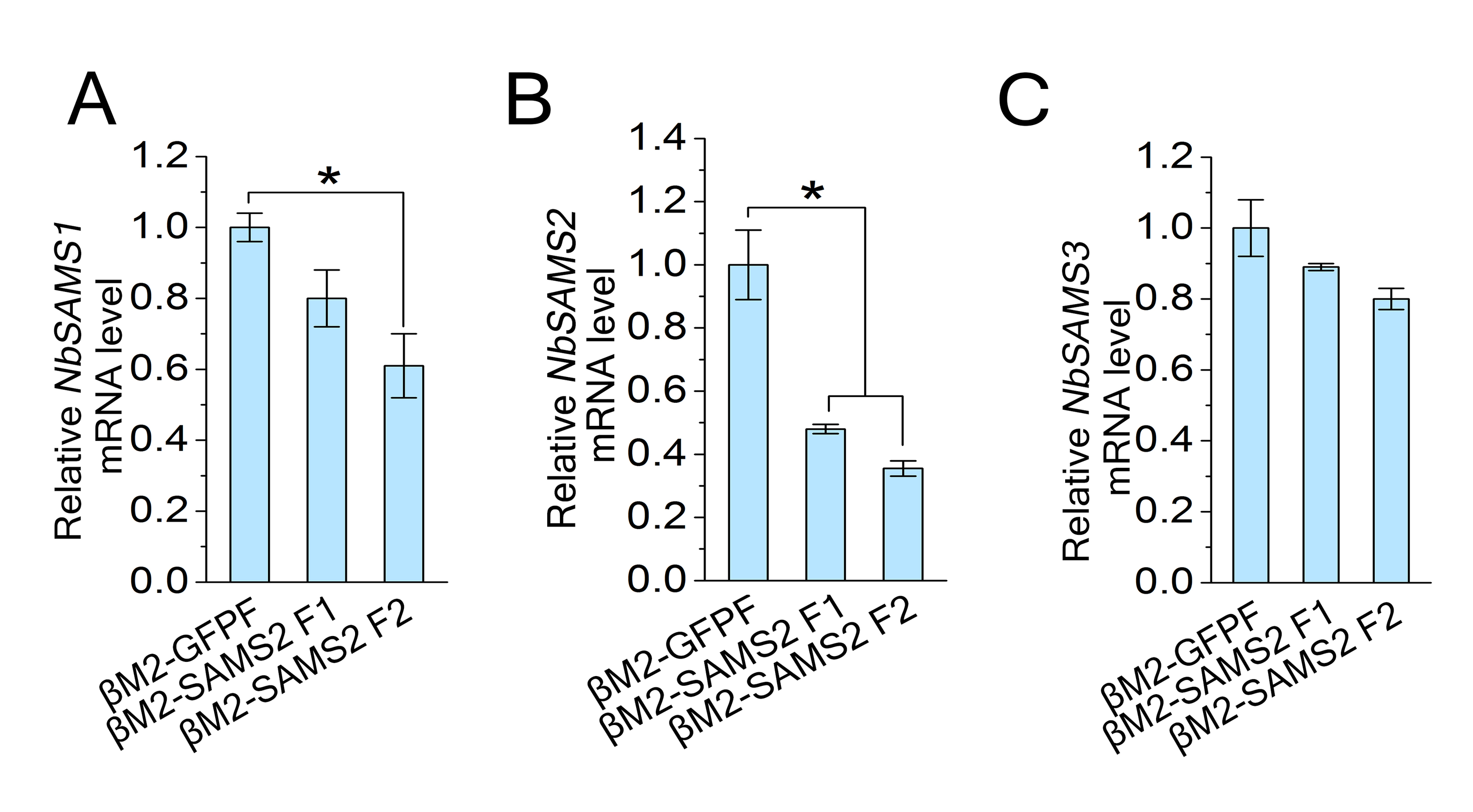

Supplement: S8 Fig — (A-C) mRNA levels of NbSAMS1, NbSAMS2 and NbSAMS3 in NbSAMS2 silenced plants respectively. Silencing was performed by using CLCuMuV as a helper virus. Real-time RT-PCR was performed using gene-specific primers. eIF4α was used as an internal control. Values represent means ± SE from three independent experiments. (*p<0.05). (TIF) [file ppat.1007282.s008.tif]

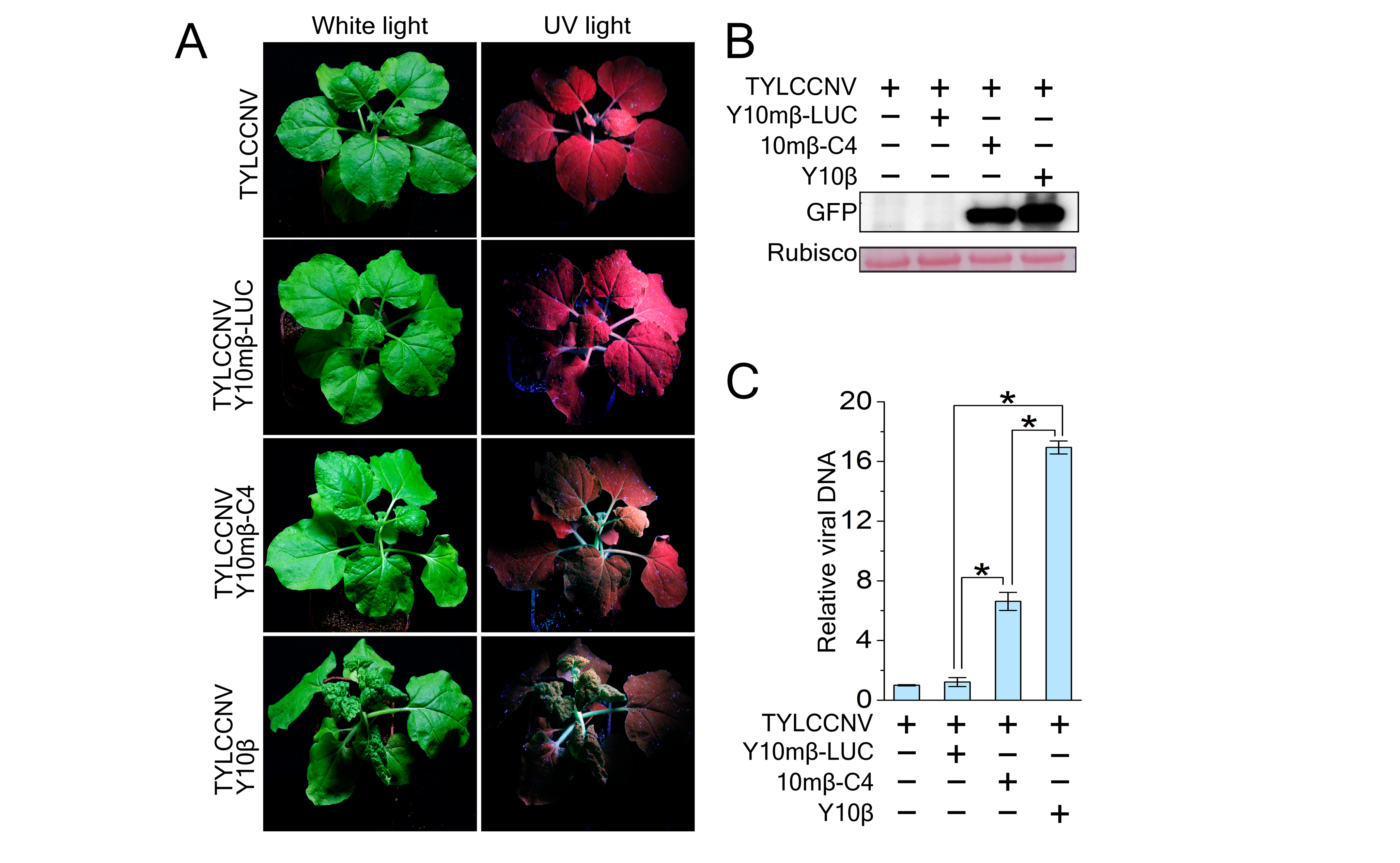

Supplement: S9 Fig — (A) TYLCCNV-based C4 expression reversed expression of a GFP transgene. N. benthamiana 16c-TGS plants were inoculated with TYLCCNV, TYLCCNV+ Y10mβ-mGFP, TYLCCNV+ Y10mβ-C4 or TYLCCNV+Y10β (as positive control). Plants were photographed under UV light at 14 dpi. (B) Western blot assay of GFP accumulation in inoculated plants. GFP protein level was assessed by anti-GFP antibody. Ponceau Red stained of Rubisco was used as a protein loading control. (C) The expression of C4 increased TYLCCNV DNA accumulation. Real-time PCR analysis of V1 gene from TYLCCNV was used to determine viral DNA level. Values represent means ± SE from three independent experiments. (TIF) [file ppat.1007282.s009.tif]

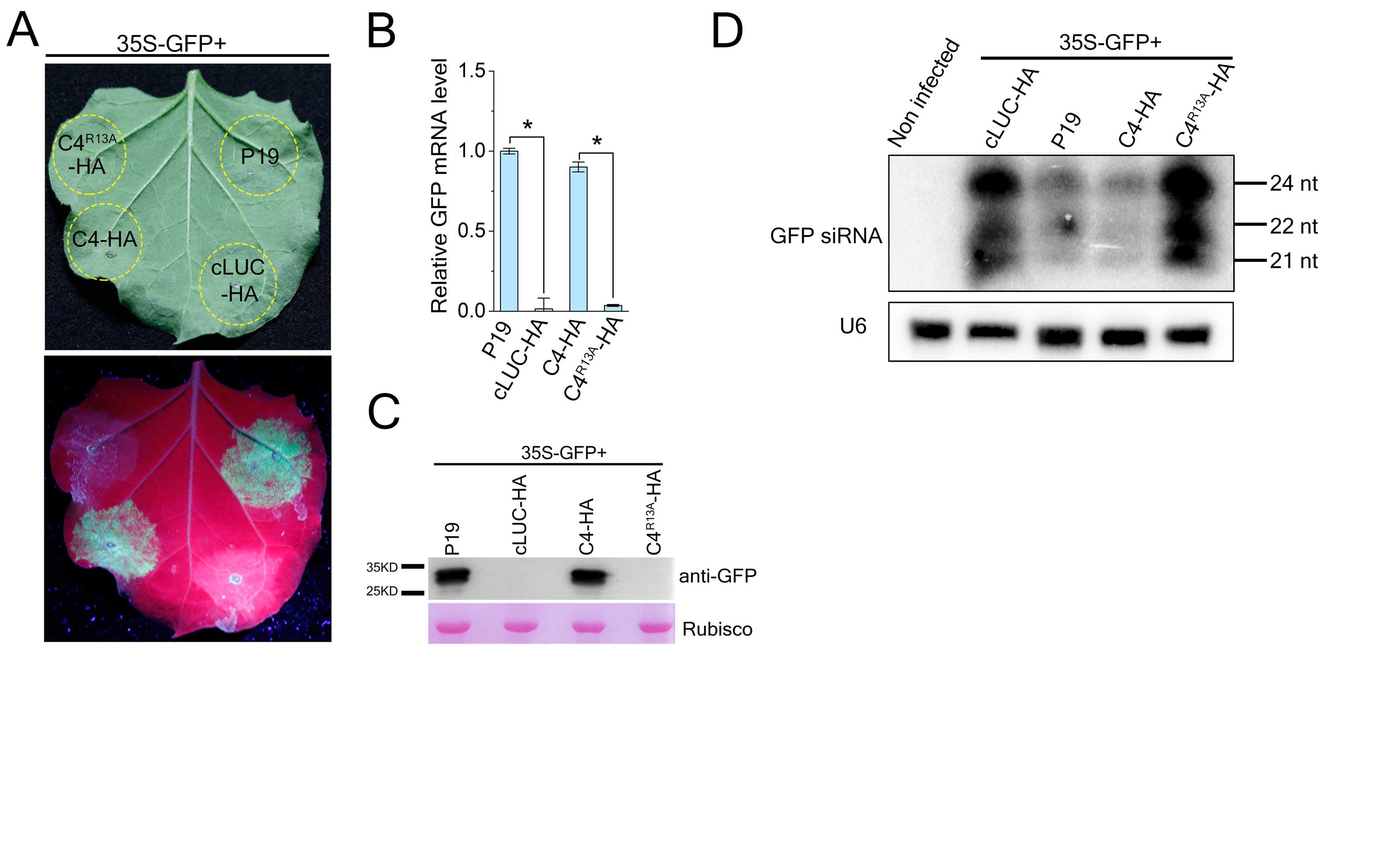

Supplement: S10 Fig — (A) GFP fluorescence in leaves of N. benthamiana plants transiently expressing 35S-GFP together with indicated suppressors. (B) Real-time RT-PCR showed relative GFP mRNA levels of leaves of 16-TGS plants inoculated as indicated. Values represent means ± SE from three independent experiments. (*p<0.05). (C) Western blot assay of GFP accumulation in inoculated plants shown in (A). GFP protein level was assessed by anti-GFP antibody. Ponceau Red Stained Rubisco was used as a protein loading control. (D) Small RNA gel blot analyses of GFP silencing in agroinfiltrated leaf samples. [γ- 32P] CTP-labeled GFP or U6 oligonucleotides were used as probes in the small RNA blots. The sizes of the 21-, 22- and 24-nt RNAs are indicated to the right of the small RNA panel. (TIF) [file ppat.1007282.s010.tif]
