# Supplementary material for: Cotton Leaf Curl Multan virus C4 protein suppresses both transcriptional and post-transcriptional gene silencing by interacting with SAM synthetase
Source: PLoS Pathog. 2018 Aug 29;14(8):e1007282. doi: 10.1371/journal.ppat.1007282 (PMC6133388; doi:10.1371/journal.ppat.1007282)
Supplement: S1 Table — (DOCX) [file ppat.1007282.s011.docx]

**Supplemental Table 1**

**Supplemental Table 1. Primers sequences Used in Vector Construction and PCR Analysis.**

| Name | Sequence | Restriction site | Used for |
| --- | --- | --- | --- |
| *AscI-NbSAMS2*-345-F1 | TCTGGCGCGCCGACTGTCAAGGCCCTCAAGCCTAAAG | *Asc1* | βM2-*SAMS2* F1 |
| *XbaI-NbSAMS2*-345-R1 | TTATCTAGATACTGCATTTCTCATTAGATAAATGAACAC | *Xba1* | βM2-*SAMS2* F1 |
| *AscI-NbSAMS2*-345-F2 | TCTGGCGCGCCAACCTATCTAAACAATGTTATTTTTTTG | *Asc1* | βM2-*SAMS2* F2 |
| *XbaI-NbSAMS2*-345-R2 | TTATCTAGACTCTGGATCTTGTTCTAGGCAAGCGTCAAG | *Xba1* | βM2-*SAMS2* F2 |
| *AscI-LUC*-345-F | TCTGGCGCGCCGAAGACGCCAAAAACATAA | *Asc1* | βM2-*LUC* |
| *XbaI-LUC-*345-R | TTATCTAGAGTTGAGCAATTCACGTTCATTAT | *Xba1* | βM2-*LUC* |
| *XbaI-LUC*-345-F | TTATCTAGAGAAGACGCCAAAAACATAA | *Xba1* | Y10mβ-LUC |
| *BamHI-LUC-*345-R | GCGGATCCGTTGAGCAATTCACGTTCATTAT | *BamHI* | Y10mβ-LUC |
| *XbaI-CLCuMuV C4-F* | TTATCTAGAATGGGAGCCCTCATCTCCATGT | *Xba1* | Y10mβ-C4 |
| *BamH1-CLCuMuV C4-R* | GCGGATCCCTAGTTCCTTAATGACTCTAAGAG | *BamHI* | Y10mβ-C4 |
| qeIF4a-F | GCTTTGGTCTTGGCACCTACTC |  | Real-time PCR |
| qeIF4a-R | TGCTCGCATGACCTTTTCAA |  | Real-time PCR |
| qCLCuMuV V1-F | ACAACAGGCATGGACAAACA |  | Real-time PCR |
| qCLCuMuV V1-R | CCAATACGATGGGTCAAACC |  | Real-time PCR |
| qTYLCCNV V1-F | GTAACGGATGTTACCCGTGG |  | Real-time PCR |
| qTYLCCNV V1-R | GTCCGCCAGTAACCGTAGAA |  | Real-time PCR |
| q*NbSAMS1*-F | GTTCTTGACGCTTGCCTT |  | Real-time PCR |
| q*NbSAMS1*-R | CACATCTTCAGAGACAAACCC |  | Real-time PCR |
| q*NbSAMS2*-F | ACAGAAGGTTATTGTGGGCT |  | Real-time PCR |
| q*NbSAMS2*-R | GTATGGTCACGAGTTTGGG |  | Real-time PCR |
| q*NbSAMS3*-F | AAGACTCGCAAGCAGTTGG |  | Real-time PCR |
| q*NbSAMS3*-R | CACAGACTCGGAGGTAAATAGG |  | Real-time PCR |
| qGFP-F | AGAGGGTGAAGGTGATGCAA |  | Real-time PCR |
| qGFP-R | TTCCCGTCGTCCTTGAAGAA |  | Real-time PCR |
| LIC1-CLCuMuV C4-F | CGACGACAAGACCGTAACCATGGGAGCCCTCATCTCCATGT |  | C4-nLUC  C4-GFP, C4-N50-GFP  C4-nYFP  C4-HA |
| CLCuMuV C4 LIC2-R | GAGGAGAAGAGCCGTCGGTTCCTTAATGACTCTAAGAGCCTCTG |  | pC4-nLUC, pC4 ^R13A^-nLUC, pC4-GFP, pC4-N50-GFP, pC4^R13A^-GFP, pC4-nYFP, pC4^R13A^-nYFP, pC4-HA, pC4^R13A^-HA, pPVX-C4-HA, pPVX-C4^R13A^-HA |
| C4-N50 LIC2-R | GAGGAGAAGAGCCGTCGAGTCCTTGACATCTGAGCTTGAT |  | pC4-N50-GFP |
| LIC1-C4-C50-F | CGACGACAAGACCGTGACCATGACATCGACAAGGACGGGGA |  | pC4-N50-GFP |
| LIC1-CLCuMuV C4^R13A^-F | CGACGACAAGACCGTAACCATGGGAGCCCTCATCTCCATGTGCTCATCCAGTTCGgcgGCAAAC |  | pC4^R13A^-GFP, pC4^R13A^-nYFP,  pC4 ^R13A^-nLUC  pC4^R13A^-HA |
| LIC2-HA-R | CGACGACAAGACCGTAACCATGTACCCATACGACGTCCC |  | pPVX-C4-HA, pPVX-C4^R13A^-HA |
| *BamHΙ*-C4-F | GCGGATCCATGGGAGCCCTCATCTCCATGT | *BamHI* | pC4-His |
| *XhoΙ*-C4-R | CGCTCGAGGTTCCTTAATGACTCTAAGAGCCTCTG | XhoΙ | pC4-His, pC4^R13A^-His |
| *BamHΙ*-C4^R13A^-F | GCGGATCCATGTGCTCATCCAGTTCGgcgGCAAAC | *BamHI* | pC4^R13A^-His |
| *NdeΙ*-C4-F | GCCATATGATGGGAGCCCTCATCTCCATGT | NdeΙ | pGST-C4 |
| *XhoΙ*-C4-R | GCCTCGAGGTTCCTTAATGACTCTAAGAGCCTCTG | XhoΙ | pGST-C4, pGST-C4 ^R13A^ |
| *NdeΙ*-C4^R13A^-F | GCCATATGATGTGCTCATCCAGTTCGgcgGCAAAC | NdeΙ | pGST-C4 ^R13A^ |
| *NdeΙ*-*NbSAMS2*-F | GCCATATGATGCGTTACTAGTATGCTCTTAAATTTGATCAG | XhoΙ | pGST- *NbSAMS2* |
| *XhoΙ*-*NbSAMS2*-R | GCCTCGAG TTATTAAGCTTTTGGCTTGAGGA | NdeΙ | pGST- *NbSAMS2* |
| *HindIII*-A-F | CGGAAGCTTAGATTTGCATTTAAATTATG | HindIII | pCLCuMuV-C4^R13A^ |
| C4^R13A^-R | TCGTGCAGATGAGTTTGCAgcCGAACTGGAT |  | pCLCuMuV-C4^R13A^ |
| C4^R13A^-F | ATCCAGTTCGgcGGCAAACTCATCTGCACGA |  | pCLCuMuV-C4^R13A^ |
| *XbaI*-A-R | GCAAATCTAGACAGGATCTTTCAGGAG | XbaI | pCLCuMuV-C4^R13A^ |
| *XbaI*-A-F | GCTCTAGAAAGATCCTGTCTAGATTTGC | XbaI | pCLCuMuV-C4^R13A^ |
| *KpnI*-A-R | CGGGGTACCAATCAAAGTACAGCACAGG | KpnI | pCLCuMuV-C4^R13A^ |
| *LIC1-NbSAMS1* -F | GAGGAGAAGAGCCGTCGTTACTAGTTCAAATTATTCTTGGATCA |  | pcLUC-*NbSAMS1* |
| *NbSAMS1* LIC2-R | GAGGAGAAGAGCCGTCGTTATTACCGGTTTTGTAAGTGTCAAC |  | pcLUC-*NbSAMS1* |
| *LIC1-NbSAMS2*-F | CGACGACAAGACCGTAACCATGCGTTACTAGTATGCTCTTAAATTTGATCAG |  | pcLUC-*NbSAMS2*  pHA-*NbSAMS2*  pcYFP- *NbSAMS2* |
| *NbSAMS2* LIC2-R | GAGGAGAAGAGCCGTCGTTATTATTAAGCTTTTGGCTTGAGGA |  | pcLUC-*NbSAMS2*  pHA-*NbSAMS2*  pcYFP-*NbSAMS2* |
| *LIC1-NbSAMS3*-F | GAGGAGAAGAGCCGTCGTTACTAGTATGTACTAGTATATAGTTTGGGT |  | pcLUC-*NbSAMS3* |
| *NbSAMS3* LIC2-R | GAGGAGAAGAGCCGTCGTTATTACCTAGCAAGACCATTGGCAA |  | pcLUC-*NbSAMS3* |
| *LIC1-35S PRO-200-F* | GAGGAGAAGAGCCGTCGTTACTAGCATGGAGTCAAAGATTCAAATAGAG |  | pTRV2-35S PRO |
| *35S PRO-200-LIC2-R* | GAGGAGAAGAGCCGTCGTTTGTTGAAAAGTCTCAATTGCCCT |  | pTRV2-35S PRO |
| *35S BIF-F* | GAAAAGGAAGGTGGTTTTTATAAATG |  | 35S bif seq |
| *35S BIF-R* | TCAATAAAAATATCACATCAATCCACT |  | 35S bif seq |
| *Tnt1 BIF-F* | GTGATGGGGTTTAGTTTTTTTAAAT |  | Tnt1 bif seq |
| *Tnt1 BIF-R* | TTACTCTTTATTTTCTCTCTTTTATTAATA |  | Tnt1 bif seq |
